# Role of Mex67-Mtr2 in the Nuclear Export of 40S Pre-Ribosomes

**DOI:** 10.1371/journal.pgen.1002915

**Published:** 2012-08-30

**Authors:** Marius B. Faza, Yiming Chang, Laura Occhipinti, Stefan Kemmler, Vikram G. Panse

**Affiliations:** 1 Institute of Biochemistry (IBC), ETH Zurich, Zurich, Switzerland; 2 MLS Program, Life Science Zurich Graduate School, Zurich, Switzerland; University of California Berkeley, United States of America

## Abstract

Nuclear export of mRNAs and pre-ribosomal subunits (pre40S and pre60S) is fundamental to all eukaryotes. While genetic approaches in budding yeast have identified *bona fide* export factors for mRNAs and pre60S subunits, little is known regarding nuclear export of pre40S subunits. The yeast heterodimeric transport receptor Mex67-Mtr2 (TAP-p15 in humans) binds mRNAs and pre60S subunits in the nucleus and facilitates their passage through the nuclear pore complex (NPC) into the cytoplasm by interacting with Phe-Gly (FG)-rich nucleoporins that line its transport channel. By exploiting a combination of genetic, cell-biological, and biochemical approaches, we uncovered an unanticipated role of Mex67-Mtr2 in the nuclear export of 40S pre-ribosomes. We show that recruitment of Mex67-Mtr2 to pre40S subunits requires loops emanating from its NTF2-like domains and that the C-terminal FG-rich nucleoporin interacting UBA-like domain within Mex67 contributes to the transport of pre40S subunits to the cytoplasm. Remarkably, the same loops also recruit Mex67-Mtr2 to pre60S subunits and to the Nup84 complex, the respective interactions crucial for nuclear export of pre60S subunits and mRNAs. Thus Mex67-Mtr2 is a unique transport receptor that employs a common interaction surface to participate in the nuclear export of both pre-ribosomal subunits and mRNAs. Mex67-Mtr2 could engage a regulatory crosstalk among the three major export pathways for optimal cellular growth and proliferation.

## Author Summary

Eukaryotic pre-ribosomal subunits (pre40S and pre60S) are one of the largest RNA containing cargos that are transported from the nucleus through the nuclear pore complex (NPC) into the cytoplasm. While genetic approaches in budding yeast have identified *bona fide* export factors for pre60S subunits, little is known regarding nuclear export of pre40S subunits. The conserved transport receptor Mex67-Mtr2 (TAP-p15 in humans) binds mRNAs and pre60S subunits in the nucleus, and facilitates their passage through the NPC, by interacting with Phe-Gly (FG)-rich nucleoporins that line its transport channel. Here, we report an additional role of Mex67-Mtr2. We show that Mex67-Mtr2 binds pre40S subunits *via* loops present on its NTF2-like domains and that the C-terminal FG-rich nucleoporin interacting UBA-like domain within Mex67 contributes to the transport of pre40S subunits to the cytoplasm. Remarkably, the same loops of Mex67-Mtr2 also contribute to the nuclear export of pre60S subunits and mRNAs. Thus, the transport receptor Mex67-Mtr2 could employ a common interaction surface to engage a regulatory crosstalk among the three major export pathways.

## Introduction

All living cells expend a significant proportion of their cellular energy to manufacture ribosomal subunits [1]. To construct ribosomal subunits, eukaryotic cells assemble >70 ribosomal proteins (r-proteins) with four different ribosomal RNA (rRNA) species [2], [3]. Transcription machineries (RNA polymerases I, II and III) are co-ordinated to ensure high efficiency and accuracy of ribosome production; Pol-I and Pol-III synthesize the rRNAs destined for 60S (25S, 5.8S, 5S rRNA) and 40S (18S rRNA) subunits, whereas Pol-II transcribes the mRNAs for the ribosomal proteins. >200 non-ribosomal factors, also termed *trans*-acting factors, aid the assembly and maturation of eukaryotic pre-ribosomal subunits [2], [3].

Our current understanding of eukaryotic ribosome assembly and intracellular transport has been shaped mainly by a combination of genetic, cell-biological and proteomic approaches applied to the model organism budding yeast. The precursor 35S rRNA produced by RNA Pol I transcription of rDNA repeats in the nucleolus is co-transcriptionally modified and associates with small subunit r-proteins and *trans*-acting factors to form the 90S particle [4]–[6]. The 90S contains mostly small subunit r-proteins but not large subunit r-proteins or *trans*-acting factors involved in 60S biogenesis [7]. Cleavage of the precursor 35S rRNA releases the pre40S particle and permits the remaining pre-rRNA to associate with large subunit r-proteins and pre60S biogenesis factors to form pre60S particles [7]. The pre40S and pre60S particles hereafter follow independent biogenesis and transport pathways. Pre40S particles undergo few compositional changes as they travel through the nucleoplasm [7], [8]. In contrast, pre60S subunits associate with ∼100 *trans*-acting factors along their biogenesis pathway and therefore undergo dynamic compositional changes as they travel through the nucleoplasm towards the NPC [9]. The stripping of *trans*-acting factors by diverse energy-consuming enzymes (ATP-dependent RNA helicases, AAA-ATPases, ABC-ATPases, GTPases) is thought to induce sequential reduction of compositional complexity resulting in export competence [10], [11].

Export competent pre40S and pre60S subunits are passaged separately through NPCs by shuttling export receptors. This is accomplished by transient interactions between export factors bound to pre-ribosomal subunits and Phe-Gly (FG)-repeat nucleoporins that line the transport channel of the NPC [12]–[14]. Transport factors include Ran-dependent exportins that interact with adaptor proteins on the pre-ribosomal subunits and *trans*-acting factors that can directly interact with FG-rich nucleoporins. The essential exportin Xpo1 (Crm1 in humans) recognizes leucine-rich nuclear export sequences (NESs) present in diverse export cargos [15] including both pre60S and pre40S subunits and mediates their nuclear export [9], [16]–[21]. Nmd3 is the only known essential NES-containing adaptor for pre60S particles that recruits Xpo1 in a RanGTP-dependent manner [16], [17]. Three Xpo1/Crm1 interacting *trans*-acting factors (Ltv1, Dim2 and hRio2) have been suggested to act as export adaptors for pre40S subunits [22]–[24], but whether they function as *bona fide* export adaptors *in vivo* is unclear [12], [24], [25]. An essential adaptor protein that recruits Xpo1 to pre40S particles has yet to be identified. Genetic studies in budding yeast have uncovered *trans*-acting factors that serve as export factors (Arx1 and Ecm1) for pre60S subunits [26]–[28]. The shuttling HEAT-repeat containing *trans*-acting factor Rrp12 was shown to promote nuclear export of both pre40S and pre60S subunits [29]. These karyopherin-like factors directly interact with the FG-rich hydrophobic meshwork of the transport channel, thereby allowing pre-ribosomal subunits to efficiently overcome the permeability barrier of the NPC [14], [26], .

Recently, the yeast heterodimeric mRNA transport factor Mex67-Mtr2 (TAP-p15 in humans) was shown to function as an export receptor for pre60S subunits [14]. Mex67-Mtr2 is structurally unrelated to karyopherins and does not rely directly on the RanGTP gradient. However, like karyopherins, Mex67-Mtr2 binds FG-rich nucleoporins and promotes nuclear export of mRNAs and pre60S subunits [14], [30]. Mex67, the large subunit of the heterodimer contains an amino-terminal (N) domain, a leucine-rich repeat (LRR) domain, a nuclear transport factor 2 (NTF2)-like middle domain and a carboxy-terminal ubiquitin associated (UBA-like) domain [31], [32]. The N and LRR domains can directly bind mRNAs or recruit mRNA binding adaptor proteins such as Yra1 [33]–[35]. Mtr2 is structurally related to NTF2, an import factor for RanGDP [36] and forms a functional heterodimer with the NTF2-like middle domain of Mex67 [34], [37]. Structural analysis revealed loops on the NTF2-like domains of Mex67-Mtr2 [38], [39] that contribute to pre60S binding *in vivo and in vitro* [14]. The loops also contribute to the recruitment of Mex67-Mtr2 to the Nup84 complex, an important structural unit of the NPC. This interaction is crucial for nuclear export of mRNAs, but not pre60S and pre40S subunit export [30]. Both the NTF2-like domains of Mex67-Mtr2 and the C-terminal UBA-like domain of Mex67 can directly bind FG-rich nucleoporins and therefore promote translocation of bound cargos through the NPC [14], [30], [34], [40]–[42].

Large-scale tandem affinity purification (TAP) combined with sensitive mass spectrometry in budding yeast revealed a co-enrichment of Slx9 with Enp1-TAP and Tsr1-TAP that purify both 90S and pre40S particles [43]. Subsequent studies implicated Slx9 in early rRNA processing steps, however its role in early pre40S maturation was not explored [44]. A rationally directed screen to identify novel factors involved in ribosome biogenesis/export in budding yeast showed that the *slx9*Δ mutant accumulates the 40S reporter S2-GFP in the nucleus [45]. Northern hybridization performed to investigate defects in rRNA processing in the *slx9*Δ mutant revealed an accumulation of 20S rRNA in the *slx9*Δ mutant, but not the early 35S precursor rRNA [45]. These observations have implicated Slx9 in late maturation steps in the 40S biogenesis pathway. Here, a genetic screen aimed at uncovering the role of Slx9 in the 40S maturation pathway unexpectedly revealed a role for Mex67-Mtr2 in pre40S subunit export. Together with previous studies, this study identifies Mex67-Mtr2 as a unique transport receptor that functions in the nuclear export of both pre-ribosomal subunits and mRNAs.

## Results

### Slx9 associates with pre-ribosomal particles in the 40S maturation pathway

**Figure 1:**
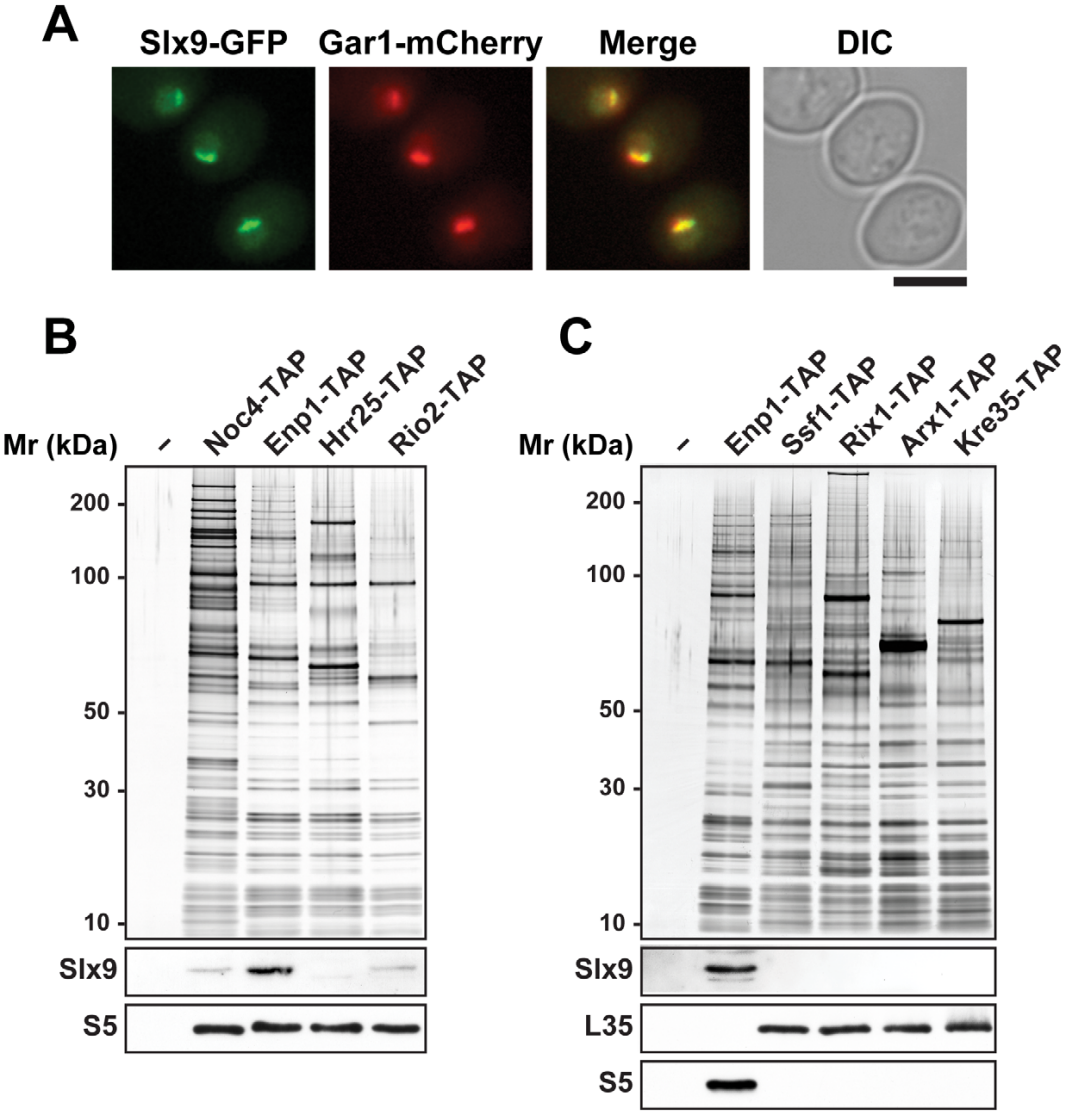
Slx9 associates with pre-ribosomal particles in the 40S maturation pathway. (A) Sub-cellular localization of Slx9 was determined by fluorescence microscopy from cells expressing Slx9-GFP and Gar1-mCherry. Bar = 5 µm. (B) Slx9 associates with pre-ribosomes in the 40S maturation pathway. (C) Slx9 does not co-enrich with pre60S subunits. (B and C) Pre-ribosomal particles in the 40S and 60S maturation pathway were purified using the indicated TAP-tagged bait proteins. The Enp1-TAP purification served as a positive control for the Slx9 blot. The calmodulin-sepharose eluates were analysed on a NuPAGE 4–12% gradient gel followed by silver staining. Western blotting was performed using antibody against Slx9. The small subunit ribosomal protein S5 and large subunit ribosomal protein L35 served as loading controls for the pre40S and pre60S subunit purifications, respectively.

Large-scale proteomic approaches in budding yeast revealed co-enrichment of Slx9 with bait proteins that purify both 90S and pre40S pre-ribosomal particles [43]. Affinity purified ProteinA-Slx9 co-enriches mainly 20S rRNA and low levels of precursor 35S rRNA [44]. Sucrose gradient sedimentation showed that Slx9 co-peaks with the 40S pool, and to lesser extent with heavier fractions [45]. To directly investigate association of the nucleolar localized Slx9 (Figure 1A) [46] with pre-ribosomal particles, we purified Noc4-TAP that purifies the early 90S, Enp1-TAP that purifies both 90S and early pre40S subunits, and two late pre40S subunits Hrr25-TAP and Rio2-TAP [7], [8]. Co-enrichment of Slx9 with the purified pre-ribosomes was investigated by Western blotting using α-Slx9 antibody. These analyses revealed that Slx9 co-enriches mainly with Enp1-TAP, that purifies both 90S and pre40S particles (Figure 1B). The Western signal for Slx9 is specific, since it was absent in Enp1-TAP isolated from an *slx9*Δ mutant (Figure S1A). Only a weak co-enrichment of Slx9 was seen in the 90S (Noc4-TAP) and the late pre40S particle purified using Rio2-TAP (Figure 1B). No association of Slx9 was detected with early to late pre-ribosomal particles in the 60S maturation pathway (Figure 1C). We conclude that Slx9 transiently associates with early 40S pre-ribosomes.

### Slx9 is required for proper pre40S nuclear export

**Figure 2:**
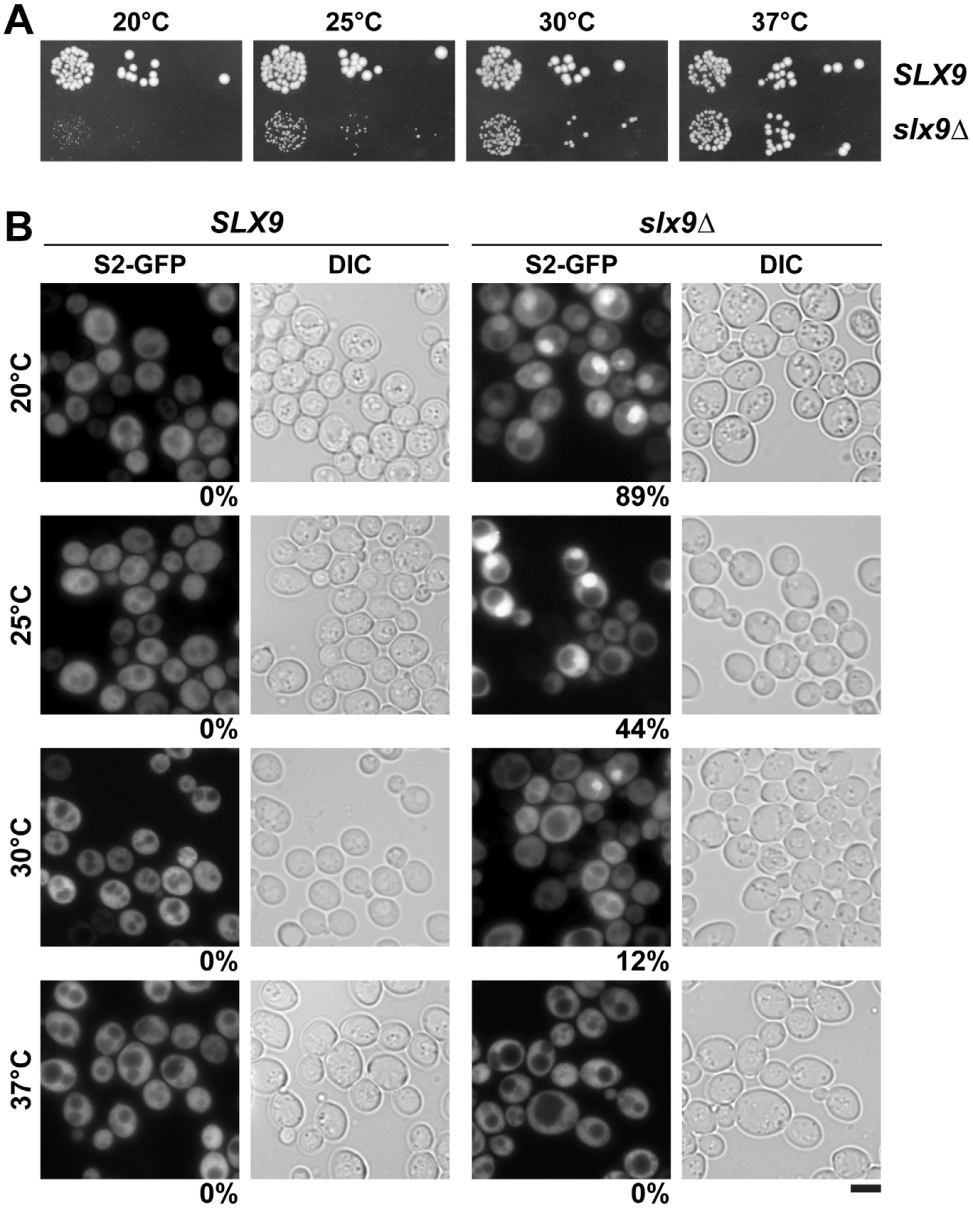
Slx9 is required for proper nuclear export of 40S pre-ribosomes. (A) The *slx9*Δ mutant is impaired in growth at lower temperatures. *SLX9* and *slx9*Δ cells were spotted in 10-fold serial dilutions on YPD plates and grown at indicated temperatures for 2–7 days. (B) The *slx9*Δ strain is impaired in pre40S subunit export. The *SLX9* and the *slx9*Δ mutant expressing the 40S reporter S2-GFP were grown at indicated temperatures. Percentage of cells showing nuclear accumulation of S2-GFP is indicated below each picture panel. Bar = 5 µm.

To investigate the role of Slx9 in the 40S maturation pathway, we generated a yeast mutant strain that is deficient for *SLX9* (*slx9*Δ) by disrupting the endogenous *SLX9* gene encoded by the open reading frame in wild-type (WT) diploid cells. Tetrad analysis yielded two spores with WT growth rates and two spores with a slow-growth phenotype at 25°C, which carry the *SLX9* deletion. We found that growth of the *slx9*Δ mutant is impaired at 20°C, 25°C and 30°C, as determined by the size of single colonies. At 37°C, the *slx9*Δ mutant grew nearly like WT cells (Figure 2A).

**Figure 3:**
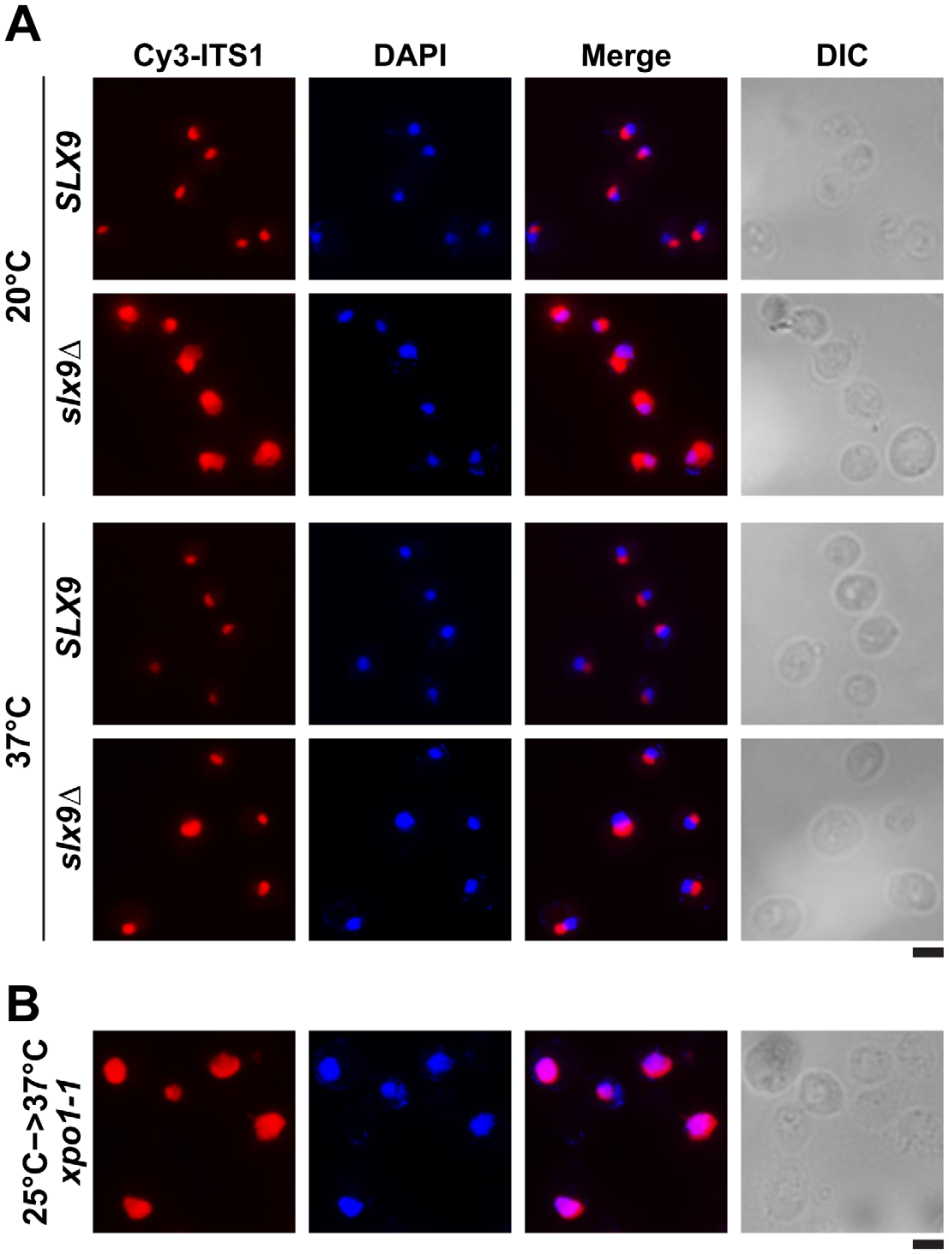
20S rRNA accumulates in the nucleoplasm in the *slx9*Δ mutant. 20S rRNA accumulates in the nucleoplasm. The *SLX9* and *slx9Δ* strains were grown to mid-log phase at the indicated temperatures and the localization of 20S rRNA was analysed by FISH using a Cy3-labeled oligonucleotide complementary to the 5′ portion of ITS1 (red). Nuclear and mitochondrial DNA was stained with DAPI (blue). The temperature sensitive *xpo1-1* mutant that accumulates pre40S subunits in the nucleoplasm served as positive control. The *xpo1-1* mutant was grown at 25°C (permissive temperature) and shifted to 37°C (restrictive temperature) for 4 h, prior to fixing cells for performing FISH. Bar = 5 µm.

Next, we investigated the localization of the 40S reporter S2-GFP in the *slx9*Δ mutant at different temperatures. At 20°C, where growth of *slx9*Δ cells is significantly impaired (Figure 2A), we found that 89% of *slx9*Δ cells showed nuclear accumulation of S2-GFP (Figure 2B). At 37°C, where the *slx9*Δ mutant grows nearly like WT cells (Figure 2A), no nuclear accumulation of S2-GFP was observed (Figure 2B). At intermediate temperatures 25°C and 30°C, 44% and 12% of the *slx9*Δ cells showed nucleoplasmic accumulation of S2-GFP, respectively (Figure 2B). We wondered whether the nucleoplasmic localization of S2-GFP in the *slx9*Δ mutant is a direct consequence of accumulating pre40S subunits. To address this, *in vivo* localization of the 5′ portion of the internal transcribed spacer 1 (ITS1), present within 20S rRNA, was monitored by fluorescence *in situ* hybridization (FISH). In WT cells, due to rapid nuclear export of pre40S subunits, Cy3-ITS1 (red) is seen in the nucleolus, but not in the nucleoplasm (DAPI, blue) (Figure 3A) [47]. Upon nuclear exit of pre40S subunits, the 5′ portion of ITS1 is efficiently degraded by the nuclease Xrn1 after cytoplasmic cleavage of the 20S rRNA into mature 18S rRNA [47], [48]. At 20°C, where 89% of the *slx9*Δ cells exhibit nuclear accumulation of S2-GFP, a strong increase in nucleoplasmic signal of Cy3-ITS1 was observed from the merge of DAPI and Cy3-ITS1 fluorescence as compared to *SLX9* cells (Figure 3A). The nucleoplasmic accumulation of Cy3-ITS1 in *slx9*Δ cells is similar to that observed in the *xpo1-1* strain, in which the nuclear export of pre40S subunits is impaired (Figure 3B) [47]. At 37°C, where *slx9*Δ cells did not show nuclear accumulation of S2-GFP (Figure 2B), Cy3-ITS1 was not detected in the nucleoplasm (Figure 3A).

Previously, a large-scale visual screen reported that the *slx9*Δ mutant is impaired in poly-(A)^+^RNA export [49]. We sought to analyse the extent of inhibition of poly-(A)^+^RNA export in the *slx9*Δ mutant, in comparison to the temperature sensitive *mex67-5* cells [31]. The *mex67-5* mutant cells after 30 min incubation at non-permissive temperature (37°C) revealed a massive nuclear accumulation of poly-(A)^+^ RNA in >95% of cells, whereas the *slx9*Δ mutant did not show such a dramatic phenotype (Figure S2A). Contrary to the previous report, only <1% of *slx9*Δ cells showed nuclear accumulation of poly-(A)^+^ RNA in the temperature range between 20–37°C (Figure S2B). Further, the *slx9*Δ mutant constructed in the W303 and RS453 backgrounds also showed nuclear accumulation of poly-(A)^+^ RNA in <1% cells, in the temperature range between 20–37°C (data not shown), suggesting that the discrepancy with the previous report might not be due to strain background differences. However, in agreement with earlier findings, the *slx9*Δ mutant was impaired only in pre40S (Figure 2B and Figure 3), but not pre60S subunit export (Figure S3A) [45].

Thus, the slow growth phenotype of the *slx9*Δ mutant between 20–30°C co-relates with impaired pre40S subunit export.

### Over-expression of Mex67-Mtr2 rescues pre40S export defect of the *slx9*Δ mutant

**Figure 4:**
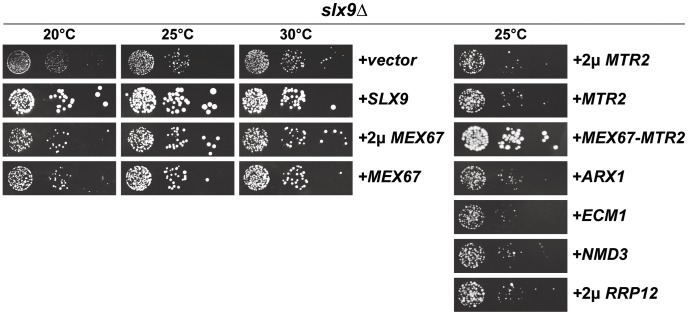
Over-expression of Mex67-Mtr2 rescues impaired growth of the *slx9*Δ mutant. The *slx9*Δ strain was transformed with the indicated plasmids, spotted in 10-fold serial dilutions on SD plates and grown at the indicated temperatures for 2–7 days.

Nucleoplasmic accumulation of pre40S subunits as judged by the small subunit reporters S2-GFP and Cy3-ITS1 indicated that Slx9 contributes to late pre40S subunit maturation/export step(s). To gain insight into the role of Slx9 in the 40S maturation/export, we resorted to a genetic screening approach. A high-copy suppressor screen was performed with the aim of identifying genes that suppress the impaired growth of the *slx9*Δ mutant. The *slx9*Δ strain was transformed with a multi-copy (2μ) plasmid library and grown at 25°C. Plasmid recovery was performed from fast growing suppressor colonies. Sequence analysis revealed that, in addition to Slx9, the mRNA and pre60S export receptor Mex67 partially rescued the slow growth of *slx9*Δ mutant at 25°C, as determined by the size of single colonies (Figure 4, left panel). Notably, Mex67 expressed from a single copy (CEN) plasmid was sufficient to partially rescue the impaired growth of *slx9*Δ mutant (Figure 4, left panel). However, Mtr2, the functional partner of Mex67, expressed alone from either CEN or 2μ plasmids did not rescue the slow growth of the *slx9*Δ mutant (Figure 4, right panel). As determined by the slightly bigger size of single colonies compared to Mex67 alone, expression of Mex67-Mtr2 from CEN plasmids in the *slx9*Δ mutant, resulted in a better rescue of the slow growth of the *slx9*Δ strain at 25°C (Figure 4, right panel). Over-expression of other pre60S export factors (Arx1, Ecm1, Nmd3 and Rrp12) from either CEN or 2μ plasmids did not rescue the slow growth phenotype of *slx9*Δ cells (Figure 4, right panel and data not shown), suggesting a specific rescue of *slx9*Δ slow growth by the export factor Mex67-Mtr2.

**Figure 5:**
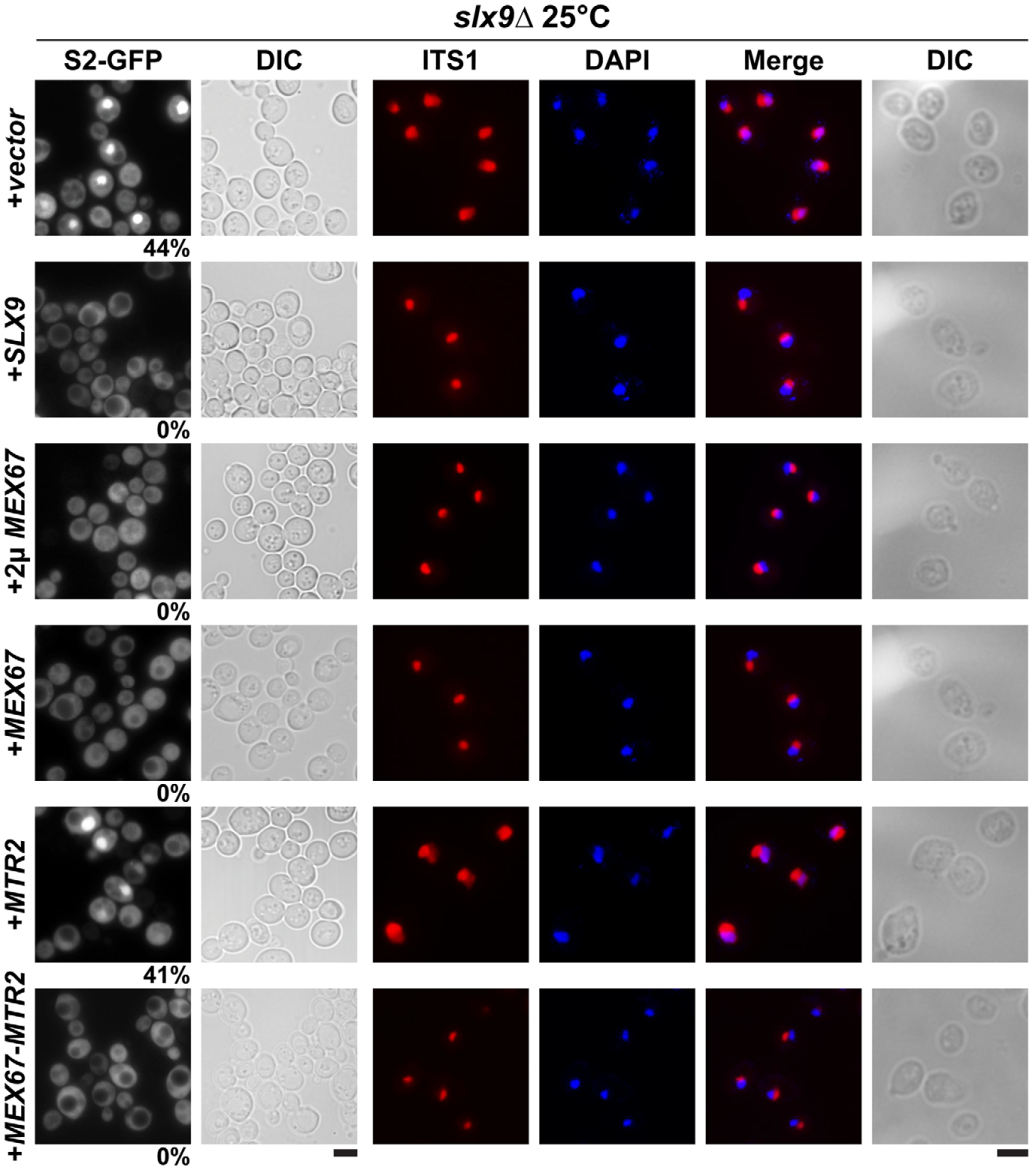
Over-expression of Mex67-Mtr2 rescues nucleoplasmic accumulation of pre40S subunits. *slx9*Δ cells containing S2-GFP were transformed with the indicated plasmids and grown on SD plates. Percentage of cells showing nuclear accumulation of the S2-GFP is indicated below each picture panel. Localization of 20S rRNA in the indicated strains was analysed by FISH using a Cy3-labeled oligonucleotide complementary to the 5′ portion of ITS1 (red). Nuclear and mitochondrial DNA was stained with DAPI (blue). Bar = 5 µm.

**Figure 6:**
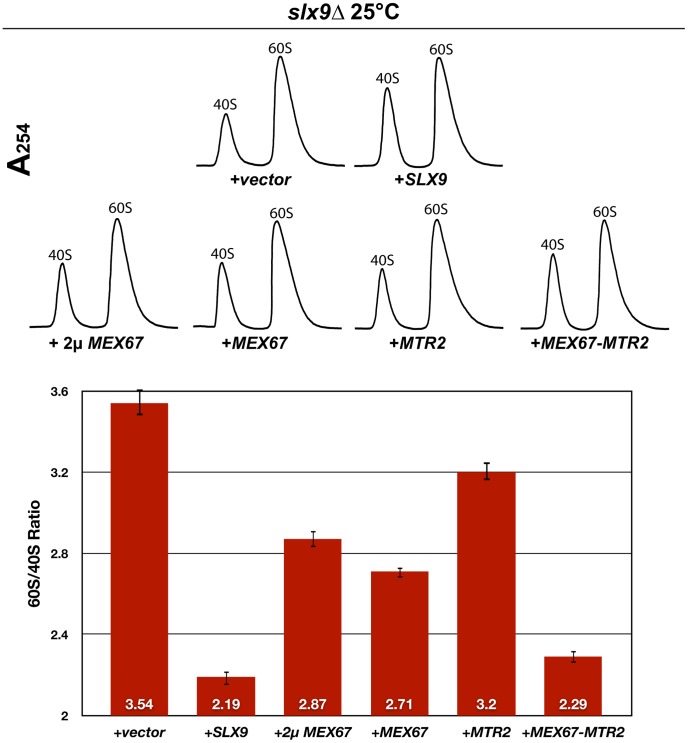
Over-expression of Mex67 and Mtr2 rescues the 60S/40S subunit imbalance of the *slx9*Δ mutant. The *slx9*Δ mutant was transformed with the indicated plasmids and grown to mid-log phase. Yeast lysates were prepared from the indicated strains in high salt conditions that dissociate the 80S and polysomes into free 40S and 60S subunits. Sucrose density gradient (7–50% sucrose) sedimentation profiles were obtained by measuring the RNA content and recorded at A_254_ after sedimentation centrifugation. A representative profile (3 independent replicates were performed) for each for indicated strain is shown. The 60S/40S ratio was determined and depicted by calculating the area under the A_254_ trace for each 60S and 40S peak using ImageJ software (version 1.42q, NIH, USA). Standard error bars are shown.

Next, we investigated whether over-expression of Mex67 could rescue the pre40S subunit export defect seen in the *slx9*Δ mutant. Over-expression of Mex67 alone (from 2μ and CEN plasmids), or Mex67-Mtr2 (from CEN plasmids), but not Mtr*2* alone (from 2μ and CEN plasmids), rescued the nucleoplasmic accumulation of both S2-GFP and Cy3-ITS1 seen in the *slx9*Δ mutant at 20°C, 25°C and 30°C (Figure 5 and data not shown). Previously, sucrose gradient sedimentation revealed a decreased abundance of 40S subunits in the *slx9*Δ mutant as compared to the WT, leading to an imbalance between free 40S and 60S subunits [45]. We quantified the 60S/40S ratio in the *slx9*Δ mutant by performing sucrose gradient sedimentation under conditions that dissociate 40S from 60S subunits [50], [51]. At 25°C, the *slx9*Δ mutant showed a 60S/40S ratio of 3.5, indicating a ∼40% reduction in the level of 40S subunits, as compared to the WT (Figure 6). We investigated whether the imbalance between 60S and 40S subunits in the *slx9*Δ mutant could be rescued by the over-expression of Mex67-Mtr2. Over-expression of Mex67 (from 2μ and CEN plasmids), that partially rescued impaired growth of *SLX9* deficient cells (Figure 4, left panel), also partially rescued the imbalance between 60S and 40S subunits observed in the *slx9*Δ mutant (Figure 6). Expression of both Mex67 and Mtr2 from CEN plasmids that resulted in a better rescue of growth of the *slx9*Δ mutant (Figure 4, right panel) restored the 60S/40S ratio closer to the WT level (Figure 6).

Together, these studies show that expression of an additional copy of Mex67-Mtr2 can compensate the requirement of Slx9 in pre40S subunit export.

### Loops of Mex67-Mtr2 and the UBA-like domain of Mex67 are crucial to rescue the pre40S export defect of the *slx9*Δ mutant

**Figure 7:**
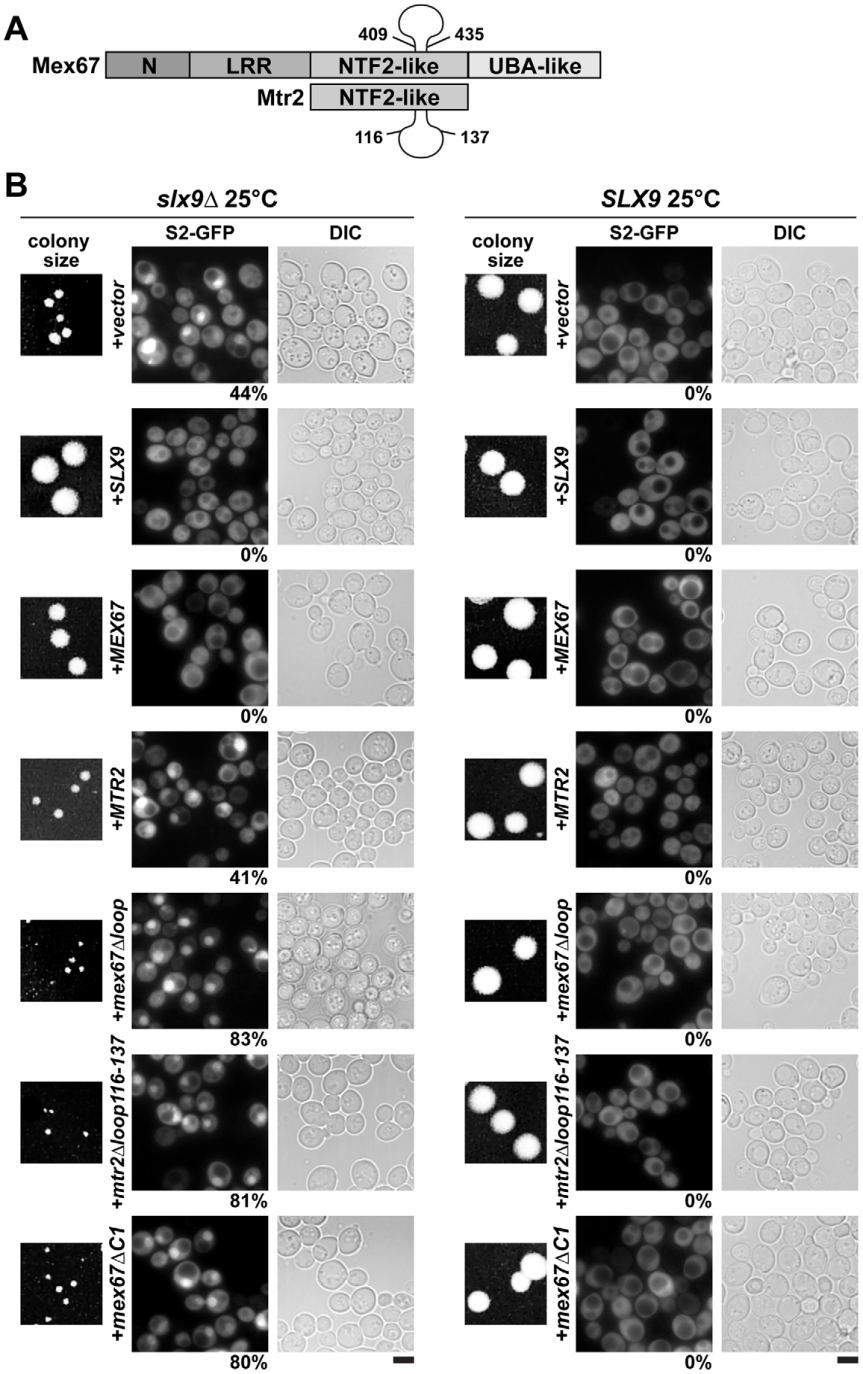
The *mex67*Δ*loop*, *mtr2*Δ*loop116-137*, and *mex67*Δ*C1* alleles are dominant negative in the *slx9*Δ mutant. (A). Schematic depicting domain organization of Mex67 and Mtr2 (not drawn to scale). Residue numbers for the alleles used in this study are indicated. (B) The *slx9*Δ (left panel) and the *SLX9* strain (right panel) were transformed with the indicated plasmids and grown on SD plates for 4 days. Localization of the S2-GFP reporter in *slx9*Δ mutant and *SLX9* cells containing the indicated plasmids were inspected by fluorescence microscopy. Percentage of cells showing nuclear accumulation of S2-GFP is indicated below each picture panel. Bar = 5 µm.

Mex67 is a modular protein that contains multiple interaction domains (Figure 7A). The N and LRR domains can directly bind mRNAs or recruit mRNA binding adaptor proteins [33]–[35]. The middle NTF2-like domain of Mex67 forms a functional heterodimer with the NTF2-like domain of Mtr2 [34], [37]. Both the middle NTF2-like domains of Mex67-Mtr2 and the C-terminal UBA-like domain of Mex67 directly bind FG-rich nucleoporins and promote translocation of bound cargoes through the NPC (Figure 7A) [34], [40]–[42]. Over-expression of Mex67-Mtr2 was reported to rescue the pre60S subunit export defect of the *nmd3*Δ*NES1* and *ecm1*Δ*arx1*Δ mutants [14], [28]. Consistent with the contribution of the loop on the NTF2-like domain of Mex67 in pre60S subunit binding, over-expression of the *mex67*Δ*loop* allele (deletion of residues 409–435 within the Mex67 loop; Figure 7A) did not rescue the pre60S export of the *nmd3*Δ*NES1* and *ecm1*Δ*arx1*Δ mutant [14], [28].

We wondered whether alleles of Mex67 and Mtr2 harbouring deletions within the different interaction domains (*mex67*Δ*loop, mex67*Δ**C1** and *mtr2*Δ*loop116-137*; Figure 7A) could rescue the impaired growth and the pre40S subunit export defect of the *slx9*Δ mutant. The *mtr2*Δ*loop116-137* allele is a shorter deletion within the loop emanating from the NTF2-like domain of Mtr2 (Figure 7A). The *mex67*Δ*loop* and *mtr2*Δ*loop116-137* alleles do not exhibit growth defects and, are not defective in the nuclear export of mRNAs, pre60S and pre40S subunits (Figure S4 and Figure S5) [14], [26], [30]. Both Mex67Δloop and Mtr2Δloop116-137 do not bind pre60S subunits and the Nup84 complex [14], [30], the interactions being important for nuclear export of pre60S subunits and mRNAs, respectively (Table 1 summarizes phenotypes and genetic interactions of *mex67*Δ*loop* and *mtr2*Δ*loop116-137* alleles). We included in our analysis the *mex67*Δ*C1* allele, a deletion of the C-terminal UBA-like domain of Mex67 (deletion of residues 525–599) that directly binds FG-rich nucleoporins and also contributes to efficient translocation of bound cargos through the NPC (Figure 7A) [34], [40]–[42]. To test whether the various alleles could rescue the impaired growth and pre40S subunit export defect of the *slx9*Δ mutant, the *slx9*Δ strain was transformed with CEN plasmids carrying *mex67*Δ*loop, mex67*Δ*C1*, and *mtr2*Δ*loop116-137* alleles. Note that the Mex67*Δ*loop, Mex67*Δ*C1 and Mtr2*Δ*loop116-137 mutant proteins are expressed similar to WT levels (Figure S1B). While expression of Mex67 partially rescued the impaired growth of the slx9Δ mutant, we found that expression of mex67Δloop, mex67Δ*C1* and *mtr2*Δ*loop116-137* alleles did not rescue the impaired growth of the *slx9*Δ mutant. Curiously, we noticed that expression of the *mex67*Δ*loop*, *mex67*Δ*C1* and *mtr2*Δ*loop116-137* alleles hampered the growth of *slx9*Δ mutant (compare single colony sizes in Figure 7B, left panel), but not *SLX9* cells (Figure 7B, right panel). Thus the *mex67*Δ*loop*, *mex67*Δ*C1* and *mtr2*Δ*loop116-137* alleles exhibit a dominant negative behaviour in the *slx9*Δ mutant, but not in the WT background.

**Table 1:** Genetic interactions and phenotypes of different loop mutants of Mex67-Mtr2.

Strains	Growth	mRNA export	60S export	40S export	Source
*mex67*Δ*loop*	–	–	–	–	[14], [26]
*arx1*Δ*mex67*Δ*loop*	very slow	–	defective	–	[26]
*mex67*Δ*loop nmd3*Δ*NES1*	inviable	inviable	inviable	inviable	[14]
*mex67Δloop nup85ΔN133*	very slow	defective	–	–	[30]
*mtr2*Δ*loop116-137*	–	–	–	–	[14], [30]
*mtr2*Δ*loop108-137* (also called *mtr2*Δloop)	very slow	–	defective	–	[14]
*mtr2*Δ*loop116-137 nup85*Δ*N133*	slow	defective	–	–	[30]
*slx9*Δ (25°C)	slow	defective <1%	–	defective 44%	this study
*slx9*Δ*mex67*Δloop	inviable	inviable	inviable	inviable	this study
*slx9*Δ*mtr2*Δ*loop116-137*	very slow	defective <1%	–	defective >90%	this study

–: not defective.

We investigated the reason for the dominant negative nature of these alleles in the *slx9*Δ mutant. The *mex67*Δ*loop* and *mtr2*Δ*loop116-137* alleles are functionally linked to both mRNA and pre60S subunit export (Table 1). Whereas, nuclear export of pre60S subunits in the single *mex67*Δ*loop* and *arx1Δ* mutants remains unaffected, the *arx1Δmex67*Δ*loop* double mutant is strongly impaired in pre60S subunit export, but not mRNA export [26]. Notably, the *mex67*Δ*loop* and the *mtr2*Δ*loop116-137* alleles, when combined with *nup85ΔN133* allele (mutant of Nup85, a component of the Nup84 complex), are impaired in mRNA export, but not pre60S subunit export (Table 1) [30]. These data led us to hypothesize that expression of *mex67*Δ*loop, mex67*Δ*C1* and *mtr2*Δ*loop116-137* alleles in the *slx9*Δ mutant induces defects in pre60S subunit and/or mRNA export, thereby further impairing growth. Surprisingly, expression of these alleles neither induced a pre60S subunit export defect in *slx9*Δ cells nor did the alleles aggravate the poly-(A)^+^RNA accumulation seen in <1% of the *slx9*Δ cells (Figure S3B and Figure S6A). Instead, the pre40S subunit export defect of *slx9*Δ cells was strongly exacerbated (Figure 7B). At 25°C, ∼44% of *slx9*Δ cells show nuclear accumulation of S2-GFP (Figure 7B). Expression of the *mex67*Δ*loop*, *mex67*Δ*C1* and *mtr2*Δ*loop116-137* alleles from CEN plasmids strongly aggravated the pre40S subunit defect of the *slx9*Δ mutant, as judged by strong nuclear accumulation of S2-GFP seen in >80% cells (Figure 7B).

We conclude that the loops on the NTF2-like domains of Mex67-Mtr2 and the C-terminal FG-rich nucleoporin interacting UBA-like domain within Mex67 play crucial roles to compensate the requirement of Slx9 in pre40S subunit export.

### Mex67-Mtr2 genetically interact with factors involved in pre40S subunit biogenesis and nuclear export

**Figure 8:**
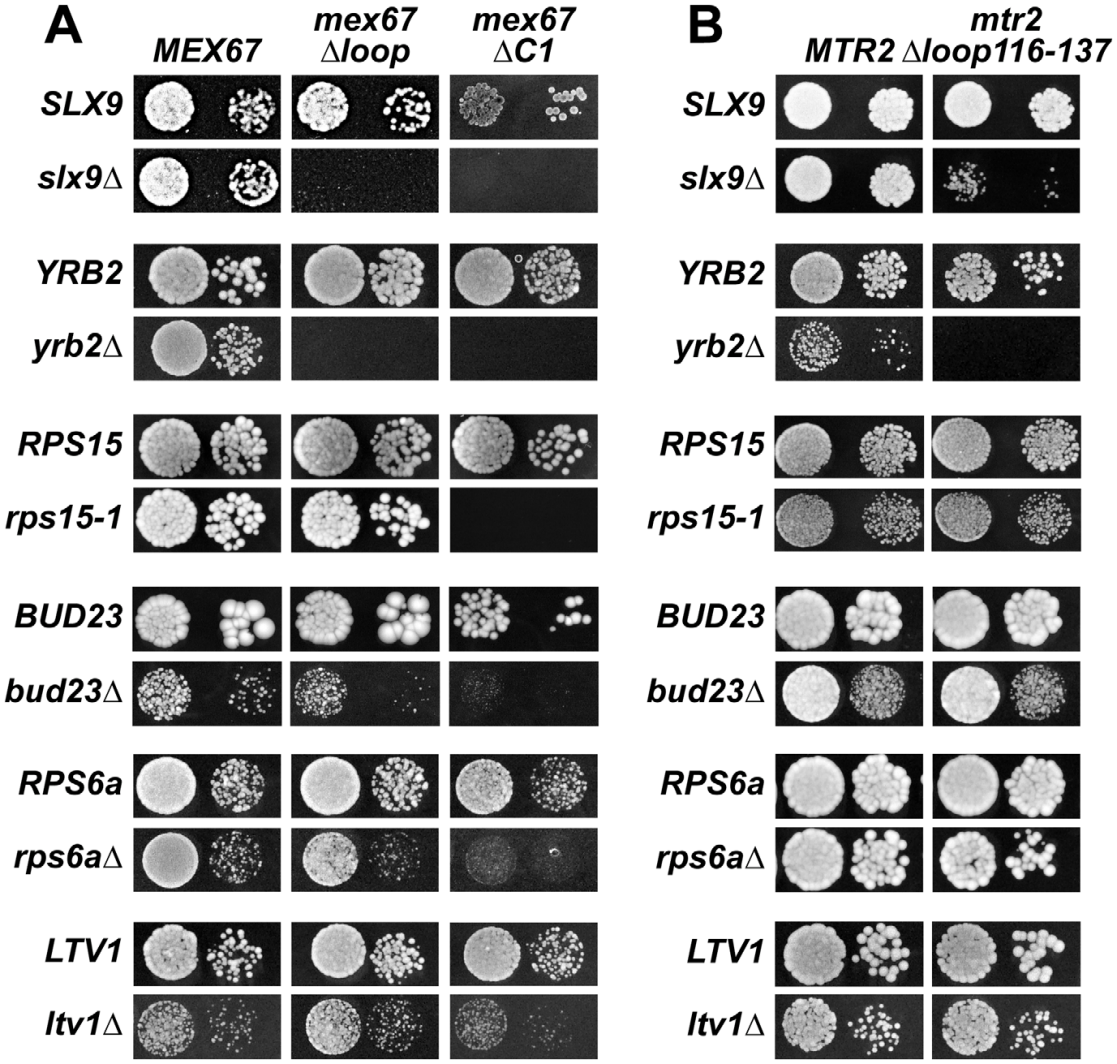
Mex67 and Mtr2 genetically interact with factors required for proper pre40S subunit export. (A and B) *mex67*Δ*loop*, *mex67ΔC1* and *mtr2Δloop116-137* alleles are synthetic lethality (*sl*) or synthetic enhancement (*se*) when combined with the indicated *slx9*Δ, *yrb2*Δ, *rps15-1*, *bud23*Δ, *rps6a*Δ and *ltv1*Δ mutants. Strains carrying the WT and mutant alleles were spotted in 10-fold serial dilutions on 5-FOA (SD) plates (when *sl*) or YPD plates (when *se*) and grown at 20–25°C for 3–9 days.

**Figure 9:**
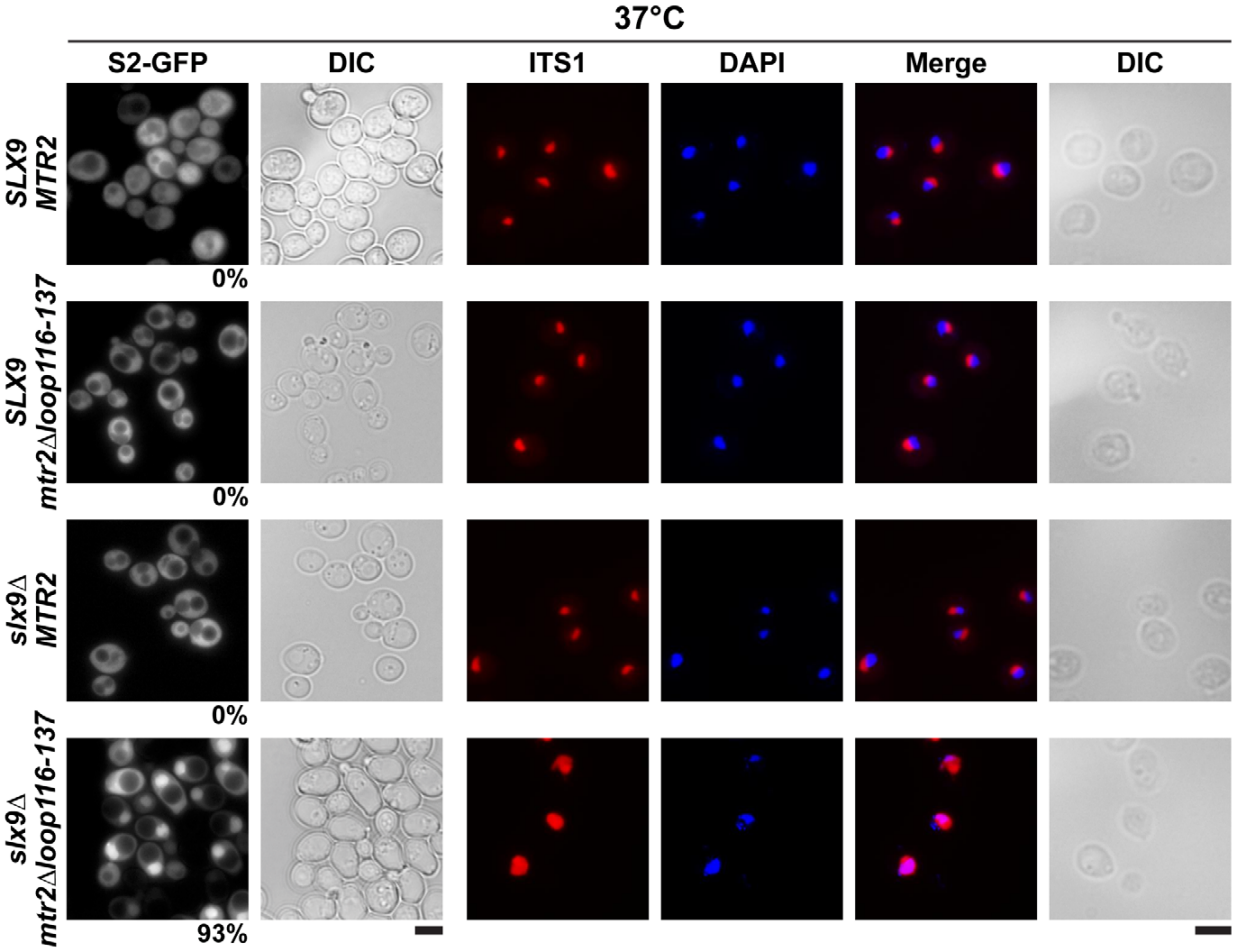
The synthetically enhanced *mtr2Δloop116-137slx9*Δ double mutant is impaired in pre40S subunit export. The indicated strains containing S2-GFP were grown at 37°C. Percentage of cells showing nuclear accumulation of S2-GFP is indicated below each picture panel. Localization of 20S rRNA in the indicated strains was analysed by FISH using a Cy3-labeled oligonucleotide complementary to the 5′ portion of ITS1 (red). Nuclear and mitochondrial DNA was stained with DAPI (blue). Bar = 5 µm.

The above data led us to test genetic interactions between Mex67-Mtr2, and Slx9. We found that the *mex67*Δ*loop and mex67*Δ*C1* alleles when combined with the *slx9*Δ mutant were synthetic lethal (Figure 8A). Remarkably, the *mtr2*Δ*loop116-137* allele when combined with the *slx9*Δ mutant was strongly synthetically enhanced (Figure 8B). We investigated whether the *slx9Δmtr2*Δ*loop116-137* strain was impaired in mRNA, pre40S and pre60S subunit export. We found that >90% of the *slx9Δmtr2*Δ*loop116-137* double mutant exhibited strong nucleoplasmic accumulation of S2-GFP and Cy3-ITS1 in the temperature range between 25–30°C (data not shown). Strikingly, at 37°C, where the *slx9*Δ and the *mtr2*Δ*loop116-137* mutants alone were not impaired in pre40S subunit export, >93% of the *slx9Δmtr2*Δ*loop116-137* double mutant showed strong nucleoplasmic accumulation of S2-GFP and Cy3-ITS1 (Figure 9). No defect in the nuclear export of pre60S subunits was observed in the *slx9*Δ*mtr2*Δ*loop116-137* strain in the temperature range between 25–37°C (Figure S3C and data not shown). Nuclear accumulation of poly-(A)^+^RNA found in <1% of the *slx9*Δ cells was not exacerbated in the double mutant *slx9Δmtr2*Δ*loop116-137* strain in the temperature range between 25–37°C (Figure S6B and data not shown). No genetic interaction was observed between Slx9 and other pre60S subunit export factors such as Nmd3, Ecm1 and Arx1 (Figure S3D).

Next, we tested further genetic interactions between Mex67-Mtr2 and factors involved in late maturation and nuclear export of pre40S subunits. The Ran binding protein Yrb2 was reported to be specifically required for proper pre40S subunit export. The *yrb2*Δ mutant exhibits nuclear accumulation of S2-GFP and reduced abundance of 40S subunits [21], [45]. We found that the *mex67*Δ*loop*, *mex67*Δ*C1* and *mtr2*Δ*loop116-137* alleles were synthetically lethal when combined with the *yrb2*Δ mutant (Figure 8). Ltv1 has been suggested as a potential adaptor for the export receptor Xpo1 [22], [52]. We found that the *ltv1*Δ mutant when combined with the *mex67*Δ*C1* allele was synthetically enhanced (Figure 8A). Growth of the *ltv1*Δ mutant was further impaired, when combined with the *mex67*Δ*loop* allele, and was unaffected when combined with the *mtr2*Δ*loop116-137* allele (Figure 8). The conserved S-adenosylmethionine methyl transferase Bud23 was reported to be required for efficient pre40S subunit export [53]. The *bud23*Δmutant exhibits nucleoplasmic accumulation of S2-GFP and Cy3-ITS1, suggesting that Bud23 acts in a late pre40S maturation/export step [53]. Notably, the enzymatic activity of Bud23 appears to be dispensable for pre40S subunit export [53]. The *bud23*Δ mutant when combined with the *mex67*Δ*loop* and *mex67*Δ*C1* alleles were synthetically enhanced, in particular the *bud23Δmex67*Δ*C1* double mutant showed strong growth impairment (Figure 8A). Growth of the *bud23*Δ mutant was unaffected when combined with the *mtr2*Δ*loop116-137* allele (Figure 8B). The ribosomal protein Rps15 has been implicated in pre40S subunit export, although its precise contribution to export step remains unclear [54], [55]. Rps15 was shown to genetically interact with Slx9 and the pre40S associated factor Bud23 [56]. Here we found that *rps15-1*, a previously reported impaired allele of Rps15 [56], when combined with the *mex67*Δ*C1* allele is synthetically lethal (Figure 8A). However, growth of *rps15-1* mutant remained unaffected when combined with the *mex67*Δ*loop* and the *mtr2*Δ*loop116-137* alleles (Figure 8A and Figure 8B). Large-scale synthetic genetic array (SGA) analysis revealed a genetic interaction between Mex67 and the small subunit ribosomal protein Rps6a [57]. For this analysis, the Guthrie and Krogan laboratories exploited a DAmP (Decreased Abundance by mRNA Perturbation) allele of Mex67. We found that the *rps6a*Δ mutant combined with the *mex67*Δ*loop* and *mex67*Δ*C1* was synthetically enhanced (Figure 8A). Growth of the *rps6a*Δ mutant was not affected when combined with the *mtr2*Δ*loop116-137* allele (Figure 8B).

At 37°C, the synthetically enhanced *slx9*Δ*mtr2*Δ*loop116-137* double mutant showed strong nucleoplasmic accumulation of Cy3-ITS1 indicating that the severe growth defect stems from impaired nuclear export of pre40S subunits, but not early rRNA processing/biogenesis defects (Figure 9). In order to directly assess whether the growth defects observed in the different synthetically enhanced mutant strains arise from either early rRNA processing/biogenesis defects or impaired nuclear export, we monitored the *in vivo* localization of ITS1 within 20S rRNA using FISH (Figure S7). Localization of ITS1 would be restricted to the nucleolus, if there were blockage in early rRNA processing/maturation steps upstream of nuclear export. The *bud22*Δ strain, that is defective in early rRNA processing steps and accumulates 35S rRNA [45], served as a control for our analyses. The Cy3-ITS1 signal is restricted to the nucleolus in the *bud22* Δmutant (Figure S7). Notably, all synthetically enhanced double mutant strains analysed exhibited a nucleoplasmic accumulation of ITS1 (Figure S7), in a manner similar to the *xpo1-1* strain (Figure 3B). Moreover, the cytoplasmic Cy3-ITS1 signal seen in the *ltv1Δ* mutant was substantially reduced in the *ltv1Δmex67*Δ*loop* and *ltv1Δmex67*Δ*C1* double mutants (Figure S7). Thus late nucleoplasmic pre40S maturation steps and/or nuclear export, not early rRNA processing, appear to be inhibited in the synthetically enhanced strains (Figure S7).

Collectively, these data point to a role of Mex67-Mtr2 in the late assembly and/or transport of pre40S subunits.

### Mex67-Mtr2 bind pre40S subunits *in vivo*

**Figure 10:**
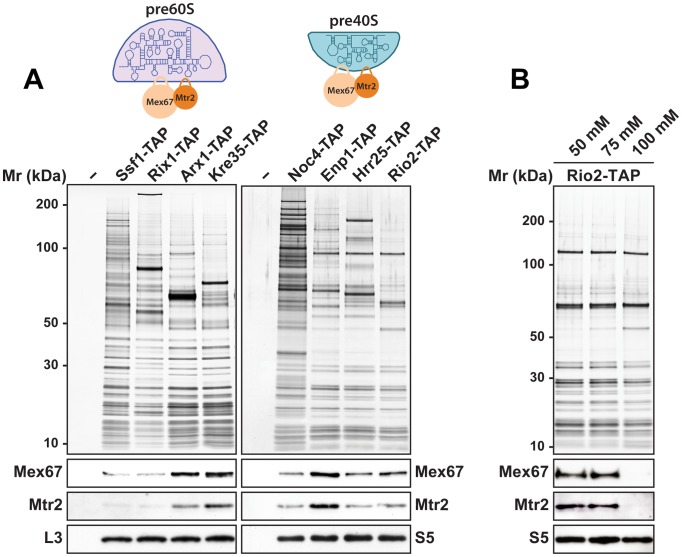
Mex67-Mtr2 co-enriches with pre40S subunits in a salt-sensitive manner. (A) Mex67-Mtr2 co-enrich with late pre60S subunits (left panel) and pre40S subunits (right panel). Tandem affinity purification (TAP) of pre60S subunits was performed *via* the indicated TAP-tagged bait proteins. (B) Association of Mex67-Mtr2 with pre40S subunits is salt sensitive. Rio2-TAP was performed using lysis buffers with different NaCl concentrations as indicated. (A and B) The calmodulin-sepharose eluates were analysed on NuPAGE 4–12% gradient gels followed by silver staining. Western blotting was performed using antibodies against Mex67 and Mtr2. The large subunit ribosomal protein L3 and the small subunit ribosomal protein S5 served as loading controls, respectively.

The rescue of impaired growth and nucleoplasmic accumulation of pre40S subunits of the *slx9*Δ mutant upon Mex67-Mtr2 over-expression and, in particular the strong synthetic interactions between Mex67-Mtr2 and the Ran-binding protein Yrb2, and the pre40S subunit associated Slx9, led us to test whether Mex67-Mtr2 co-enriches pre40S subunits. We purified early 90S (Noc4-TAP), early (Enp1-TAP) and late (Hrr25-TAP, Rio2-TAP) pre40S particles [7], [8]. As additional controls, we purified several pre60S particles at different stages of maturation in parallel. These include an early (Ssf1-TAP), an intermediate (Rix1-TAP), a late (Arx1-TAP) and a cytoplasmic pre60S particle (Kre35-TAP) [9]. The purified pre-ribosomal subunits were analysed by SDS-PAGE and Western blotting was performed using α-Mex67 and α-Mtr2 antibodies. As previously shown in [14], Mex67 and Mtr2 co-enrich with late pre60S particles (Figure 10A, left panel). Consistent with our genetic and cell-biological observations, we found that Mex67-Mtr2 co-enriches with pre40S subunits and to a lesser extent with a 90S particle (Noc4-TAP; Figure 10A, right panel). These biochemical studies show that Mex67-Mtr2 co-enriches with pre40S subunits *in vivo*. Next, we examined the nature of the interaction between Mex67-Mtr2 and pre40S subunits, by isolating the Rio2-TAP particle in different NaCl concentrations. These analyses showed that the Mex67-Mtr2 remained stably bound to pre40S subunits at 50 mM and 75 mM NaCl. Association of Mex67 and Mtr2 with Rio2-TAP was lost at 100 mM NaCl (Figure 10B). These data suggest that the interactions between Mex67-Mtr2 and pre40S subunits are likely to be driven by electrostatic interactions.

### Loops emanating from the NTF2-like domains of Mex67 and Mtr2 contribute to pre40S subunit binding

**Figure 11:**
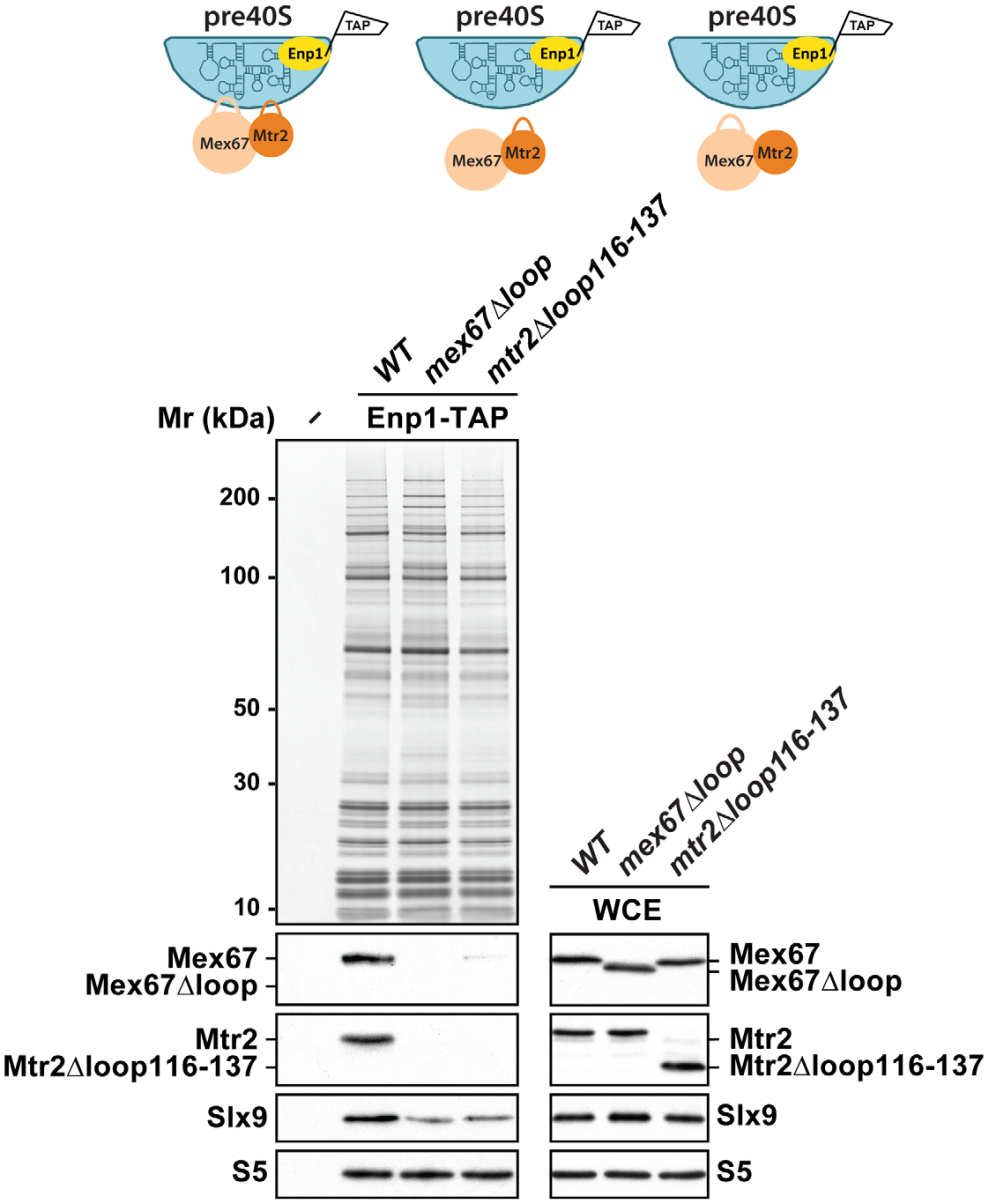
Mex67-Mtr2 bind pre40S subunits *via* loops emanating from their NTF2-like domains. Enp1-TAP was purified from *mex67*Δ*loop* and *mtr2*Δ*loop116-137* strains. The calmodulin-sepharose eluates were analysed on NuPAGE 4–12% gradient gels followed by Western blotting using antibodies against Mex67, Mtr2, and Slx9. The small subunit ribosomal protein S5 served as loading control. Whole cell extracts (WCEs) of the same strains were prepared as described in Materials and Methods and analysed by Western blotting using antibodies against Mex67, Mtr2, Slx9 and S5.

We investigated how Mex67-Mtr2 binds pre40S subunits. Two observations raised the possibility that the loops present on the NTF2-like domains of Mex67-Mtr2 contribute to pre40S subunit binding: (1) expression of the *mex67*Δ*loop and mtr2*Δ*loop116-137* alleles exacerbated the pre40S subunit export defect of the *slx9*Δ mutant and (2) *mex67*Δ*loop* and *mtr2*Δ*loop116-137* alleles genetically interact with factors required for efficient pre40S subunit export. To address whether the loops of Mex67-Mtr2 contribute to pre40S subunit binding, we purified Enp1-TAP from *mex67*Δ*loop* and *mtr2*Δ*loop116-137* strains and examined the co-enrichment of Mex67Δloop and Mtr2Δloop116-137 mutant proteins by Western blotting using α-Mex67 and α-Mtr2 antibodies (Figure 11, left panel). Note that antibodies against Mex67 and Mtr2 recognize both Mex67*Δ*loop and Mtr2*Δ*loop116-137 mutant proteins in whole cell extract (WCE) samples, respectively (Figure 11, right panel). These analyses revealed that the Mex67Δloop and the Mtr2Δloop116-137 mutant proteins fail to co-enrich with pre40S particles (Figure 11, left panel). Next, we investigated whether the association of Mex67 with pre40S subunits was dependent on the loop of Mtr2 and *vice versa*. Consistent with our genetic and cell-biological analysis, pre40S subunits affinity purified *via* Enp1-TAP from *mtr2*Δ*loop116-137* and *mex67*Δ*loop* strains fail to co-enrich Mex67 and Mtr2, respectively (Figure 11, left panel). These analyses revealed that Mex67Δloop and the Mtr2Δloop116-137 mutant proteins fail to co-enrich with pre40S particles. Similar observations were made when Rio2-TAP was purified from *mtr2*Δ*loop116-137* and *mex67*Δ*loop* strains (Figure S1C). We conclude that, like in the case of pre60S subunits and the Nup84 complex, both loops on the NTF2-like domains of Mex67-Mtr2 contribute to pre40S subunit binding.

## Discussion

How pre-ribosomal subunits efficiently overcome the permeability barrier of the NPC is poorly understood. To achieve brisk export rates, pre-ribosomal subunits recruit several transport factors at distinct sites on their surface [13], [14], [29]. Therefore, uncovering export receptors for pre-ribosomal subunits will contribute towards our current understanding to this highly conserved transport process. While genetic approaches in budding yeast have greatly aided the identification factors that participate in pre60S subunit transport, little is known regarding nuclear export of pre40S subunits.

Here, we have uncovered an unanticipated role for Mex67-Mtr2 in 40S pre-ribosome export. A genetic screen revealed that expression of an additional copy of Mex67-Mtr2 rescues the nuclear accumulation of S2-GFP and Cy3-ITS1 of the *slx9*Δ mutant (Figure 5). Mex67 and Mtr2 genetically interact with *trans*-acting factors associated with pre40S subunits and involved in their nuclear export. Different alleles of Mex67 and Mtr2 (*mex67*Δ*loop*, *mex67*Δ*C1* and *mtr2*Δ*loop116-137*) when combined with the *slx9*Δ, *yrb2*Δ, *bud23*Δ and *rps6a*Δ mutants were either synthetically lethal or enhanced (Figure 8). Moreover, at 37°C though the *slx9*Δ and *mtr2*Δ*loop116-137* mutants are not impaired in pre40S subunit export, the *slx9Δmtr2*Δ*loop116-137* double mutant exhibits strong nucleoplasmic accumulation of S2-GFP and ITS1 (Figure 9). These data are analogous to genetic interactions observed between Mex67 and factors (Nmd3, Arx1 and Ecm1) that directly participate in the nuclear export of pre60S subunits [14], [28]. Over-expression of Mex67, not the *mex67*Δ*loop* allele, was shown to rescue the pre60S subunit export defect of the *nmd3*Δ*NES1* and *ecm1*Δ*arx1*Δ mutants [14], [28]. Whereas the single mutant *arx1*Δ and *mex67*Δ*loop* are unaffected in pre60S subunit export, the *arx1*Δ*mex67*Δ*loop* double mutant is strongly impaired in pre60S subunit export [26].

The NTF2 fold is a functionally versatile domain in nuclear transport. The transport factor NTF2, that contains the NTF2 domain and functions as a homodimer, binds RanGDP in the cytoplasm and simultaneously engages in interactions with the NPC for importing RanGDP into the nucleus [36]. The NTF2-like domains of Mex67-Mtr2 exhibit loops on their surface (Figure 7A) that contribute to the interaction with double stranded regions of 5S rRNA *in vitro* [14], suggesting that Mex67-Mtr2 could interact with a structured rRNA segment on the surface of pre60S subunits. The Hurt laboratory has exploited the *mex67*Δ*loop* and *mtr2*Δ*loop116-137* alleles to uncover another physical interaction of Mex67-Mtr2 loops: the Nup84 complex [30]. Genetic and cell-biological studies revealed that this interaction is crucial for mRNA export, but not pre60S subunit export [30]. In the present study, these alleles have helped to reveal a role for Mex67-Mtr2 loops in pre40S export. Whereas an additional copy of Mex67-Mtr2 rescued the pre40S export defect of the *slx9*Δ mutant, expression of the *mex67*Δ*loop* and *mtr2*Δ*loop116-137* alleles strongly aggravated this phenotype in a dominant negative manner (Figure 7). Consistent with these genetic and cell-biological data, biochemical studies show that binding of Mex67-Mtr2 to pre40S subunits is mediated by both loops (Figure 11). Whether the loops of Mex67-Mtr2 *in vivo* interact with an exposed structured rRNA or a protein factor(s) on pre40S subunits remains to be determined. In addition to the loops, other regions of the NTF2-like fold of Mex67-Mtr2 contribute to pre60S subunit binding. Only the *mex67*Δ*loopK343E* mutant is impaired in nuclear export of pre60S subunits [14]. These data prompted us to construct alleles of Mex67 that are specifically defective in pre40S subunit export. To this end, several positively charged residues on the NTF2-like fold were mutated in *mex67*Δ*loop* (*R353E*, *K366E*, *K370E*, *K372E*, *K439E*, *K442E* and *K443E*). Unfortunately, these mutants did not complement the lethality of the *MEX67* null strain (data not shown), and hence we were unable to perform further phenotypic and functional analysis. Information regarding the precise molecular contacts between the NTF2-like folds of Mex67-Mtr2 and pre40S subunits should aid the rational design of mutants of Mex67 and Mtr2 that are specifically impaired in pre40S export.

In the mRNA export pathway, co-transcriptional recruitment of Mex67-Mtr2 to the growing nascent pre-mRNA takes place during early steps of mRNP biogenesis [58], [59]. In the 60S pathway, Mex67-Mtr2 is recruited to late export competent pre60S subunits (Figure 10A, left panel) [14]. Here, we found that Mex67-Mtr2 maximally co-enriches with early 40S pre-ribosomes isolated *via* Enp1-TAP that purifies both the 90S and early pre40S subunits (Figure 10A, right panel). Thus pre40S subunits might be competent to load the Mex67-Mtr2, perhaps after separation of the 90S into 40S and 60S precursors. Biochemical analysis revealed that the loops of Mex67-Mtr2 play a role in stable incorporation of Slx9 into early pre40S subunits (Figure 11). Notably, strong genetic interactions were observed between Mex67-Mtr2 and Slx9, which also co-enriches mainly with early pre40S subunits (Enp1-TAP; Figure 1B). These data raise the possibility that recruitment of Mex67-Mtr2 *via* the loops to pre40S subunits might be required for maturation steps that lead to the formation of export competent pre40S subunits.

The C-terminal UBA-like domain of Mex67 interacts with FG-rich nucleoporins and directly contributes to the transport of bound cargos through the NPC [34], [40]–[42]. We found that the *mex67*Δ*C1* allele did not rescue the pre40S subunit export defect of the *slx9*Δ mutant; instead, this allele exacerbated the pre40S export defect of *slx9*Δ cells (Figure 7B). Notably, growth of the *mex67*Δ*C1* allele when combined with the *slx9*Δ, *yrb2*Δ, *rps15-1*, *ltv1*Δ, *rps6a*Δ and *bud23*Δ were either synthetic lethal or strongly synthetically enhanced (Figure 8). These data indicate that the FG-rich nucleoporin interacting UBA-like domain contributes to the function of Mex67 in pre40S subunit export. How Mex67-Mtr2 is released from pre40s subunits remains to be determined. Yet unknown energy consuming factors could trigger the release of Mex67-Mtr2 from pre40S subunits [60], [61].

What could be the reason for the dominant negative nature of the *mex67*Δ*loop*, *mtr2*Δ*loop116-137* and *mex67*Δ*C1* alleles in the *slx9*Δ mutant? A functional full-length Mex67-Mtr2 becomes limiting and essential for pre40S subunit nuclear export in the *slx9*Δ mutant (Figure 5 and Figure 7). Thus one plausible explanation could be that expression of the *mex67*Δ*loop*, *mtr2*Δ*loop116-137* and *mex67*Δ*C1* alleles poisons the WT Mex67-Mtr2 by forming either Mex67-Mtr2*Δ* *loop116-137 or Mex67*Δ*loop-Mtr2 heterodimers, that cannot bind pre40S subunits (Figure 11). Alternatively, in the case of the mex67Δ* *C1* allele, a mex67*Δ*C1-Mtr2 heterodimer could be formed that cannot efficiently interact with FG-rich nucleoporins. Expression of *mex67*Δ*loop*, *mtr2*Δ*loop116-137* and *mex67*Δ*C1* alleles might therefore aggravate the pre40S subunit export defect in the *slx9*Δ mutant (not in *SLX9*) by creating non-functional Mex67-Mtr2 heterodimers that either do not bind pre40S subunits or inefficiently transport pre40S subunits through the NPC.

What could be the role of Slx9 in pre40S subunit maturation/export pathway? Pre40S subunits are exported out of the nucleus containing 20S rRNA precursor. Upon reaching the cytoplasm, the 20S rRNA is cleaved to mature 18S rRNA releasing the ITS1 fragment for degradation by the nuclease Xrn1 [47], [48]. In this study, we found a strong nuclear accumulation of pre40S subunits in the *slx9*Δ mutant as judged by the localisation of S2-GFP and the 5′ portion of ITS1 (Figure 2 and Figure 3A). The nucleoplasmic accumulation of ITS1 seen in the *slx9*Δ mutant is similar to that observed in the *xpo1-1* strain that is impaired in nuclear export of pre40S subunits (Figure 3B). Because a pre40S subunit containing 20S pre-rRNA is exported into the cytoplasm, a blockage in subunit export is expected to result in increased levels of 20S rRNA [21]. This has been observed for the *slx9*Δ mutant [45]. These data suggest a requirement of Slx9 for efficient nuclear export of the pre40S subunits. Large-scale synthetic genetic array (SGA) screens from the Krogan and Boone laboratories revealed strong genetic interactions between Slx9 and several nucleoporins (Nup57, Nup120, Nup2, Nup53, Asm4, Nup42), and integral nuclear membrane proteins that are required for NPC biogenesis (Apq12, Pom34) [57], [62]. These results indicate that Slx9 is embedded in a network of functional interactions involving the NPC. However, Slx9 did not bind the export receptor Xpo1 in presence of RanGTP *in vitro*, suggesting that it does not contain a nuclear export signal and therefore is unlikely to be an export adapter (data not shown). Slx9 may be necessary for recruitment of an export adaptor on maturing pre40S subunits. However, Mex67-Mtr2 levels on Enp1-TAP were not altered in the *slx9*Δ mutant suggesting that Slx9 might facilitate incorporation of a yet unknown export factor onto pre40S subunits (Figure S1A). While the recruitment of Mex67-Mtr2 to pre40S subunits remained unaffected in the *slx9*Δ mutant, we found that efficient recruitment of Slx9 to pre40S subunits requires Mex67-Mtr2 loops (Figure 11). These data indicate that recruitment of Mex67-Mtr2 to pre40S subunits precedes stable incorporation of Slx9. Another possibility could be that Slx9 participates in a yet unknown maturation step that renders pre40S subunits export competent. In line with this possibility, Slx9 briefly visits early pre-ribosomal particles in the 40S maturation pathway (Figure 1B). The precise role of Slx9 in pre40S subunit maturation/export pathway remains to be determined.

Previously, a high-throughput visual screen reported that the *slx9*Δ mutant is impaired in nuclear export of poly-(A)^+^ RNAs [49]. However, in our hands, <1% of the *slx9*Δ cells showed nuclear accumulation of poly-(A)^+^ RNA in the temperature range between 20–37°C (Figure S2A). This discrepancy appears not to be due to differences in strain backgrounds, since the *slx9*Δ mutant constructed in three different backgrounds (SC228c, W303 and RS453) exhibited the same phenotype (data not shown). Notably, the *mex67*Δ*loop* allele, which in combination with the *nup85*Δ*N133* mutant showed an mRNA export defect (Table 1) [30], did not further exacerbate the nuclear poly-(A)^+^RNA accumulation of the *slx9*Δ mutant (Figure S6A). Only the pre40S export defect of the *slx9*Δ mutant was strongly aggravated upon the expression of the *mex67*Δ*loop* allele (Figure 7B). Further, the nuclear accumulation of poly-(A)^+^ RNA observed in <1% of the *slx9*Δ cells was not aggravated when combined with the *mtr2*Δ*loop116-137* allele (Figure S6B). The synthetically enhanced *slx9Δmtr2*Δ*loop116-137* double mutant strain was strongly impaired only in the nuclear export of pre40S subunits (Figure 9). Finally, Slx9 does not genetically interact with factors (Sub2 and Yra1) that are specifically involved in mRNA export (Figure S2B). Altogether, these results do not support a function for Slx9 in the mRNA export pathway.

Ribosome production is one of most energy consuming processes that needs to be quickly repressed or induced in response to nutrient availability. An extensive regulatory crosstalk must exist between the mRNA and ribosome biogenesis/export pathways that ensure correct levels/stoichiometry of mRNAs and ribosomes reach the cytoplasm. Mex67-Mtr2 could co-ordinate nuclear export of pre-ribosomal particles and mRNAs. Moreover, given the vital need to rapidly transport mRNAs and pre-ribosomes to the cytoplasm, Mex67-Mtr2 could step in to perform the duty of pre40S subunit export under circumstances when either adaptors for Xpo1 or yet unknown karyopherin-like factors are not recruited to pre40S subunits. Mex67-Mtr2 could export some of the pre40S subunits *albeit* with less efficiency to keep a certain cytoplasmic pool of mature ribosomes, and thus translation, sustained in *slx9*Δ cells. How is the cellular pool of Mex67-Mtr2 fractionated to participate in the nuclear export of mRNAs, pre40S and pre60S ribosomal subunits? Unravelling the mechanism(s) by which the three transport pathways cooperate to ensure the arrival of appropriate levels of pre-ribosomal subunits and mRNAs in the cytoplasm is a challenge for the future.

## Materials and Methods

### Yeast strains and plasmids

The *Saccharomyces cerevisiae* strains used in this study are listed in Table S1. Genomic disruptions and C-terminal tagging at the genomic loci were performed as described previously [63]–[65]. Preparation of media, yeast transformations, mating, sporulation of diploids, tetrad analysis, genetic manipulations and the *slx9*Δ high-copy suppressor screen were performed according to established procedures.

Plasmids used in this study are listed in Table S2. Details of plasmid construction will be provided upon request. All recombinant DNA techniques were performed according to established procedures using *E. coli* XL1 blue cells for cloning and plasmid propagation. All cloned DNA fragments generated by PCR amplification were verified by sequencing.

### Genetic methods

Genetic interactions were tested as described previously [26]. (a) Example for a synthetic lethal interaction: the *mex67Δslx9*Δ strain containing pURA3-*MEX67* was transformed with pairs of plasmids and grown on –LeuTrp (SD) plates (media that does not select for the pURA3-*MEX67* plasmid): (1) pLEU2-*MEX67*/pTRP1-*SLX9*; (2) pLEU2-*mex67*Δ*loop*/pTRP1-*SLX9*; (3) pLEU2-*MEX67*/pTRP1-empty; (4) pLEU2-*mex67*Δ*loop*/pTRP1-empty. To score for a genetic interaction, the transformants were spotted on 5-FOA (SD). (b) Example for synthetic enhancement: the *mtr2Δslx9*Δ strain containing the pURA3-*MTR2* was transformed with the following plasmids: (1) pLEU2-*MTR2*/pTRP1-*SLX9*; (2) pLEU2-*mtr2*Δ*loop116-137*/pTRP1-*SLX9*; (3) pLEU2-*MEX67*/pTRP1-empty; (4) pLEU2-*mtr2*Δ*loop116-137*/pTRP1-empty. The *mtr2Δslx9*Δ strain containing the pURA3-*MTR2*, containing the plasmids: pLEU2-*mtr2*Δ*loop116-137* and the pTRP1-empty, grew very slowly on 5-FOA (SD) plates (as compared to the single *mtr2*Δ*loop116-137* and *slx9*Δ mutants), indicating a synthetic enhancement. The strains that grew on 5-FOA (SD) plates were subsequently spotted on YPD plates at different temperatures for analysis.

### Tandem affinity purification

Tandem affinity purification (TAP) of pre-ribosomal particles were carried out as described previously [51], [65]–[67]. All purifications were performed using the TAP lysis buffer (50 mM Tris-HCl, pH 7.5, 75 mM NaCl, 1.5 mM MgCl_2_, and 0.15% NP-40). Eluates of all TAP purifications were analysed by NuPAGE 4–12% Bis-Tris gel (Invitrogen) followed by silver staining or Western blotting.

### 40S and 60S subunit export reporter assays

The L25-GFP and S2-GFP reporter assays to analyse pre-ribosomal subunit export were performed as previously described [51], [67]. Cells were observed by fluorescence microscopy (see below). Percentages of cells exhibiting nuclear export defects (mRNA, pre40S and pre60S) reported in this study were averaged from three independently performed experiments. >200 cells were analysed for each strain indicated.

### Fluorescence *in situ* hybridization

Fluorescence *in situ* hybridization (FISH) to monitor nuclear accumulation of poly-(A)^+^RNA in different strains was carried out using Cy3-oligo (dT)^30^ as previously described [68]. Nucleoplasmic accumulation of 20S rRNA in the different strains was carried using a Cy3-labeled oligonucleotide probe (5′-Cy3-ATG CTC TTG CCA AAA CAA AAA AAT CCA TTT TCA AAA TTA TTA AAT TTC TT-3′) that is complementary to the 5′ portion of ITS1 as previously described [69].

### Sucrose gradient sedimentation

Sucrose gradient sedimentation to determine the subunit stoichiometry was performed as described previously [50], [51]. The indicated strains were grown to OD_600_ 0.8 and lysed in lysis buffer (50 mM Tris-HCl, pH 7.5, 50 mM KCl, and 1 mM DTT). The lysate was clarified by centrifugation and loaded onto 7–50% sucrose density gradient containing 50 mM Tris-HCl, pH 7.5, 50 mM KCl, and 1 mM DTT. The gradient was centrifuged at 39,000 rpm for 165 min (SW41 rotor; Beckman Coulter). Run-off polysome profiles were recorded by measuring rRNA at A_254_ using a density gradient fractionator (Teledyne).

### Fluorescence microscopy

Cells were visualized using DM6000B microscope (Leica) equipped with HCX PL Fluotar 63× and 100× 1.25 NA oil immersion objective (Leica). Images were acquired with a fitted digital camera (ORCA-ER; Hamamatsu Photonics) and Openlab software (PerkinElmer).

### Western blotting and miscellaneous

Western blot analysis was performed according to standard protocols. The following primary antibodies were used in this study: α-Slx9 (1∶1000; this study), α-Mex67 (1∶5,000; C. Dargemont, Institut Jacques Monod, France), α-Mtr2 (1∶1000; E. Hurt, University of Heidelberg, Germany), α-Nmd3 (1∶5,000, A. Johnson; University of Texas-Austin, USA), α-S3 (1∶2,000; Proteintech), α-S5 (1∶4,000; this study), α-L3 (1∶10,000; J. Warner, Albert Einstein College of Medicine, USA), α-L35 (1∶4,000, this study), and α-TAP (1∶4,000; Thermo Scientific). The secondary HRP-conjugated α-rabbit and α-mouse antibodies (Sigma-Aldrich) were used at 1∶1,000 to 1∶5,000 dilutions. Proteins were visualized using Immun-Star HRP chemiluminescence kit (Bio-Rad). Whole cell lysates were prepared by alkaline lysis of yeast cells as previously described [51].

## Supporting Information

Figure S1 Recruitment of Mex67-Mtr2 to 40S pre-ribosomes. (A) Recruitment of Mex67-Mtr2 to pre40S subunits is unaffected in the *slx9*Δ mutant. Enp1-TAP was purified from *SLX9* and *slx9*Δ strains. The purified TAP particles were analysed on NuPAGE 4–12% gradient gels followed by silver staining. Western blotting was performed using antibodies against Slx9, Mex67, Mtr2 and CBP. The small subunit ribosomal protein S5 served as loading control. (B) Whole cell extracts (WCEs) were analysed by Western blotting using antibodies against Mex67, Mtr2 and TAP tag. The bait Rio2-TAP served as loading control. (C) Rio2-TAP was purified from the *mex67*Δ*loop* and *mtr2*Δ*loop116-137* strains. The eluates were analysed on NuPAGE 4–12% gradient gels followed by Western blotting using antibodies against Mex67 and Mtr2. The small subunit ribosomal protein S5 and Rio2-CBP served as loading controls.
(TIF)

Figure S2 (A) <1% of *slx9*Δ cells exhibit nuclear accumulation of poly-(A)^+^RNA. *SLX9* and *slx9*Δ strains were grown to mid-log phase in YPD at the indicated temperatures. Localization of poly-(A)^+^RNA was performed by FISH using Cy3-labelled oligo-(dT)^30^. Nuclear and mitochondrial DNA was stained with DAPI. Percentage of cells showing nuclear accumulation of poly-(A)^+^RNA is indicated below each picture panel. The *mex67-5* strain that accumulated poly-(A)^+^RNA at 37°C served as positive control. The *mex67-5* strain was grown at 25°C, then shifted to 37°C for 1 h prior to FISH analyses. Percentage of cells showing nuclear accumulation of poly-(A)^+^RNA is indicated below each picture panel. Bar = 5 µm. (B) Slx9 does not genetically interact with factors involved in mRNA export. Growth of the *slx9*Δ mutant combined with the *sub2-85* and *yra1*Δ*RRM* alleles. The strains were spotted in 10-fold serial dilutions on YPD plates and grown at 25°C for 2–3 days.
(TIF)

Figure S3 (A) The *slx9*Δ mutant is not impaired in pre60S subunit nuclear export. *SLX9* and *slx9*Δ strains containing the 60S reporter, L25-GFP, were grown at 20°C and inspected by fluorescence microscopy. Percentage of cells that showed nuclear accumulation of the L25-GFP is indicated below each picture panel. The *yvh1Δ* mutant that accumulates the L25-GFP in the nucleoplasm in the temperature range between 20–37°C served as positive control. Bar = 5 µm. (B) Expression of *mex67*Δ*loop*, *mex67*Δ*C1* and *mtr2*Δ*loop116-137* alleles in the *slx9*Δ mutant does not induce a pre60S subunit export defect. The *slx9*Δ mutant containing the L25-GFP reporter was transformed with the indicated plasmids and grown at 25°C. The localization of L25-GFP was inspected by fluorescence microscopy. Percentage of cells showing nuclear accumulation of the L25-GFP is indicated below each picture panel. The *yvh1Δ* mutant that accumulates the L25-GFP in the nucleoplasm at 25°C served as positive control. Bar = 5 µm. (C) The synthetically enhanced *slx9Δmtr2*Δ*loop116-137* strain is not impaired in pre60S subunit export. Localization of L25-GFP in the indicated strains was inspected by fluorescence microscopy at 37°C. Percentage of cells showing nuclear accumulation of the L25-GFP is indicated below each picture panel. The *yvh1Δ* mutant that accumulates the L25-GFP in the nucleoplasm at 37°C served as positive control. Bar = 5 µm. (D) Slx9 does not genetically interact with factors involved in pre60S subunit export. Growth of the *slx9*Δ mutant combined with the *arx1Δ*, *ecm1Δ* and the *nmd3*Δ*NES1* mutants. The indicated strains were spotted in 10-fold serial dilutions on YPD plates and grown at 30°C for 2–3 days.
(TIF)

Figure S4 The *mex67*Δ*loop* and *mtr2*Δ*loop116-137* alleles do not accumulate poly-(A)^+^RNA in the nucleus. The *mex67*Δ*loop* and *mtr2*Δ*loop116-137* strains were grown at the indicated temperatures. Localization of poly-(A)^+^RNA was performed by FISH using Cy3-oligo-(dT)^30^. Nuclear and mitochondrial DNA was stained with DAPI. The *mex67-5* strain that accumulated poly-(A)^+^RNA at 37°C served as positive control. The *mex67-5* strain was grown at 25°C, then shifted to 37°C for 1 h prior to analyses. Percentage of cells showing nuclear accumulation of poly-(A)^+^RNA is indicated below each picture panel. Bar = 5 µm.
(TIF)

Figure S5 The *mex67*Δ*loop* and *mtr2*Δ*loop116-137* alleles are not impaired in pre40S and pre60s subunit nuclear export. The *mex67*Δ*loop* and *mtr2*Δ*loop116-137* strains containing S2-GFP or L25-GFP were grown at the indicated temperatures and inspected by fluorescence microscopy. Percentage of cells showing nuclear accumulation of the S2-GFP and L25-GFP is indicated below each picture panel. Bar = 5 µm.
(TIF)

Figure S6 (A) Expression of *mex67*Δ*loop*, *mex67*Δ*C1* and *mtr2*Δ*loop116-137* alleles does not exacerbate nuclear accumulation of poly-(A)^+^RNA in the *slx9*Δ mutant. The *slx9*Δ mutant containing *mex67*Δ*loop*, *mex67*Δ*C1* and *mtr2*Δ*loop116-137* alleles were grown at 25°C to mid-log phase. Localization of poly-(A)^+^RNA was performed by FISH using Cy3-labelled oligo-(dT)^30^. Nuclear and mitochondrial DNA was stained with DAPI. Percentage of cells showing nuclear accumulation of poly-(A)^+^RNA is indicated below each picture panel. The *mex67-5* strain that accumulated poly-(A)^+^RNA at 37°C served as positive control. The *mex67-5* strain was grown at 25°C, then shifted to 37°C for 1 h prior to analyses. Percentage of cells that showed nuclear accumulation of poly-(A)^+^RNA is indicated below each picture panel. Bar = 5 µm. (B) Nuclear accumulation of poly-(A)^+^RNA is not aggravated in the synthetically enhanced *slx9Δmtr2*Δ*loop116-137* strain. The indicated strains were grown to mid-log phase at 37°C. Localization of poly-(A)^+^RNA was performed by FISH using Cy3-labelled oligo-(dT)^30^. Nuclear and mitochondrial DNA was stained with DAPI. Percentage of cells showing nuclear accumulation of poly-(A)^+^RNA is indicated below each picture panel. The *mex67-5* strain that accumulated poly-(A)^+^RNA at 37°C served as positive control. The *mex67-5* strain was grown at 25°C, then shifted to 37°C for 1 h prior to analyses. Percentage of cells that showed nuclear accumulation of poly-(A)^+^RNA is indicated below each picture panel. Bar = 5 µm.
(TIF)

Figure S7 Synthetically enhanced double mutant strains accumulate ITS1 in the nucleoplasm. The indicated strains analysed in Figure 8 were grown to mid-log phase at 30°C and shifted to 20°C for 3 h. Localization of 20S rRNA was analysed by FISH using a Cy3-labeled oligonucleotide complementary to the 5′ portion of ITS1 (red). Nuclear and mitochondrial DNA was stained with DAPI (blue). Bar = 5 µm.
(TIF)

Table S1 List of plasmids used in this study.
(PDF)

Table S2 List of yeast strains used in this study.
(PDF)
