# Supplementary figures and images for: Role of Mex67-Mtr2 in the Nuclear Export of 40S Pre-Ribosomes
Source: PLoS Genet. 2012 Aug 30;8(8):e1002915. doi: 10.1371/journal.pgen.1002915 (PMC3431309; doi:10.1371/journal.pgen.1002915)

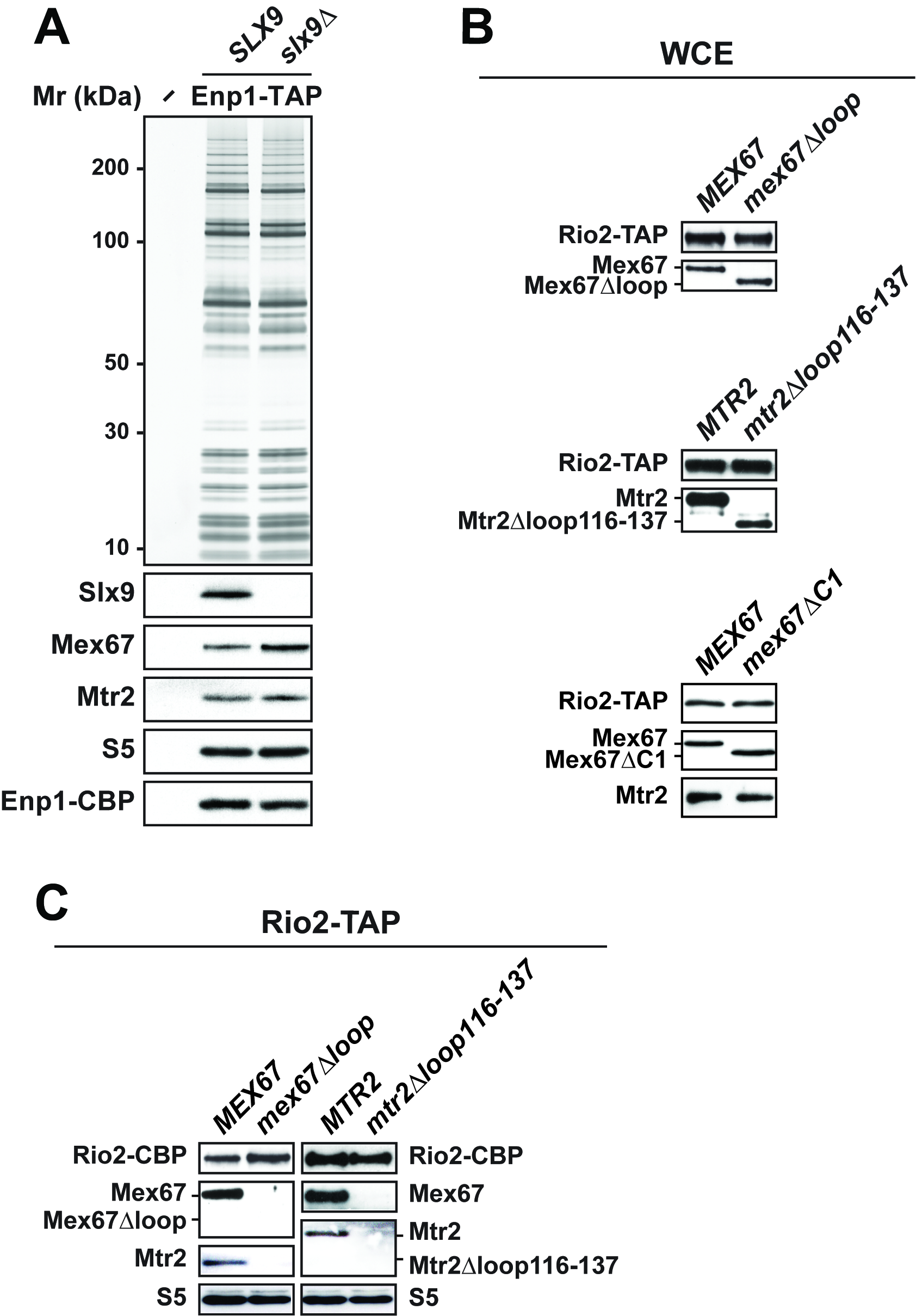

Supplement: Figure S1 — Recruitment of Mex67-Mtr2 to 40S pre-ribosomes. (A) Recruitment of Mex67-Mtr2 to pre40S subunits is unaffected in the slx9Δ mutant. Enp1-TAP was purified from SLX9 and slx9Δ strains. The purified TAP particles were analysed on NuPAGE 4–12% gradient gels followed by silver staining. Western blotting was performed using antibodies against Slx9, Mex67, Mtr2 and CBP. The small subunit ribosomal protein S5 served as loading control. (B) Whole cell extracts (WCEs) were analysed by Western blotting using antibodies against Mex67, Mtr2 and TAP tag. The bait Rio2-TAP served as loading control. (C) Rio2-TAP was purified from the mex67Δloop and mtr2Δloop116-137 strains. The eluates were analysed on NuPAGE 4–12% gradient gels followed by Western blotting using antibodies against Mex67 and Mtr2. The small subunit ribosomal protein S5 and Rio2-CBP served as loading controls. (TIF) [file pgen.1002915.s001.tif]

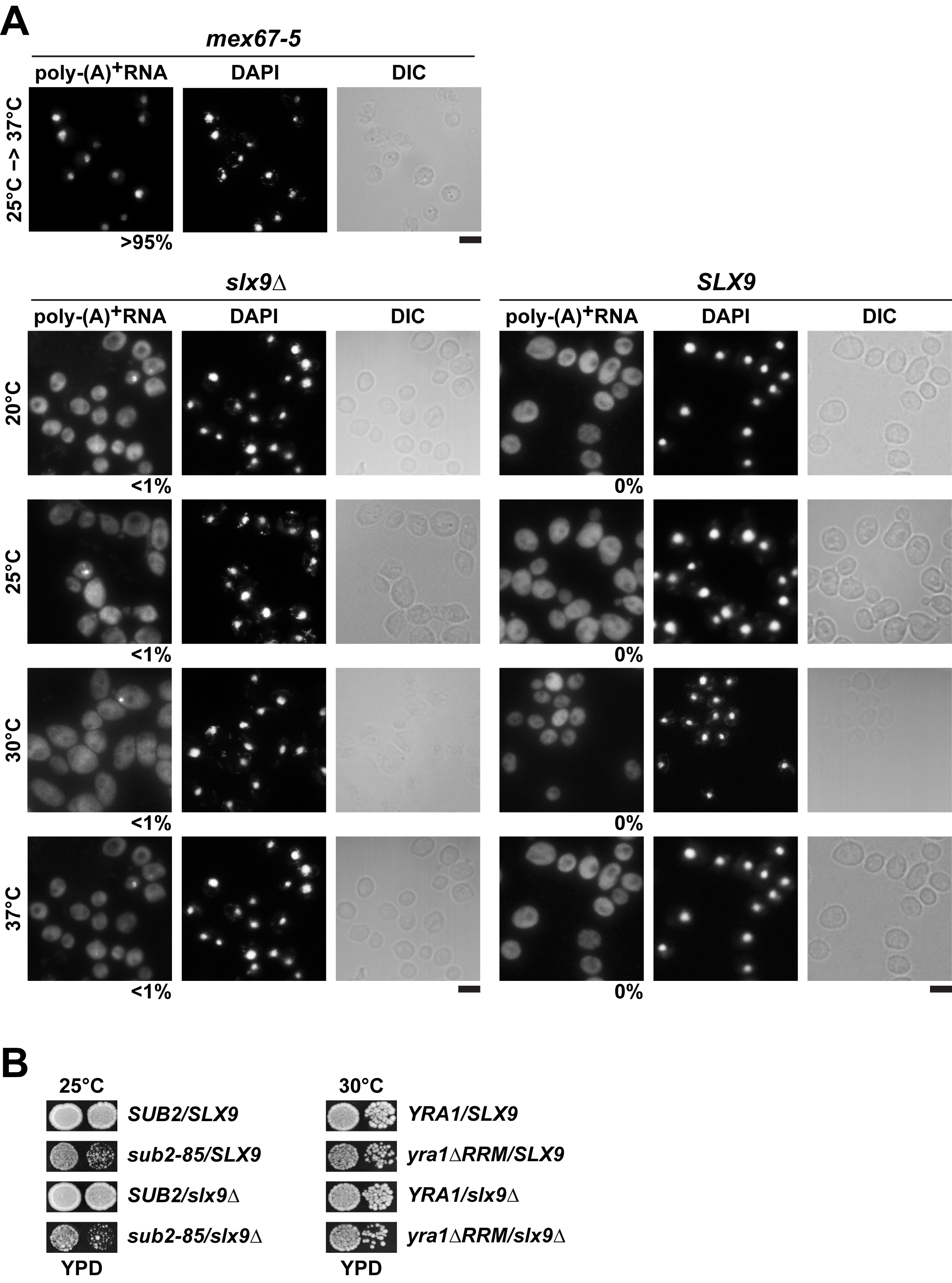

Supplement: Figure S2 — (A) <1% of slx9Δ cells exhibit nuclear accumulation of poly-(A)+RNA. SLX9 and slx9Δ strains were grown to mid-log phase in YPD at the indicated temperatures. Localization of poly-(A)+RNA was performed by FISH using Cy3-labelled oligo-(dT)30. Nuclear and mitochondrial DNA was stained with DAPI. Percentage of cells showing nuclear accumulation of poly-(A)+RNA is indicated below each picture panel. The mex67-5 strain that accumulated poly-(A)+RNA at 37°C served as positive control. The mex67-5 strain was grown at 25°C, then shifted to 37°C for 1 h prior to FISH analyses. Percentage of cells showing nuclear accumulation of poly-(A)+RNA is indicated below each picture panel. Bar = 5 µm. (B) Slx9 does not genetically interact with factors involved in mRNA export. Growth of the slx9Δ mutant combined with the sub2-85 and yra1ΔRRM alleles. The strains were spotted in 10-fold serial dilutions on YPD plates and grown at 25°C for 2–3 days. (TIF) [file pgen.1002915.s002.tif]

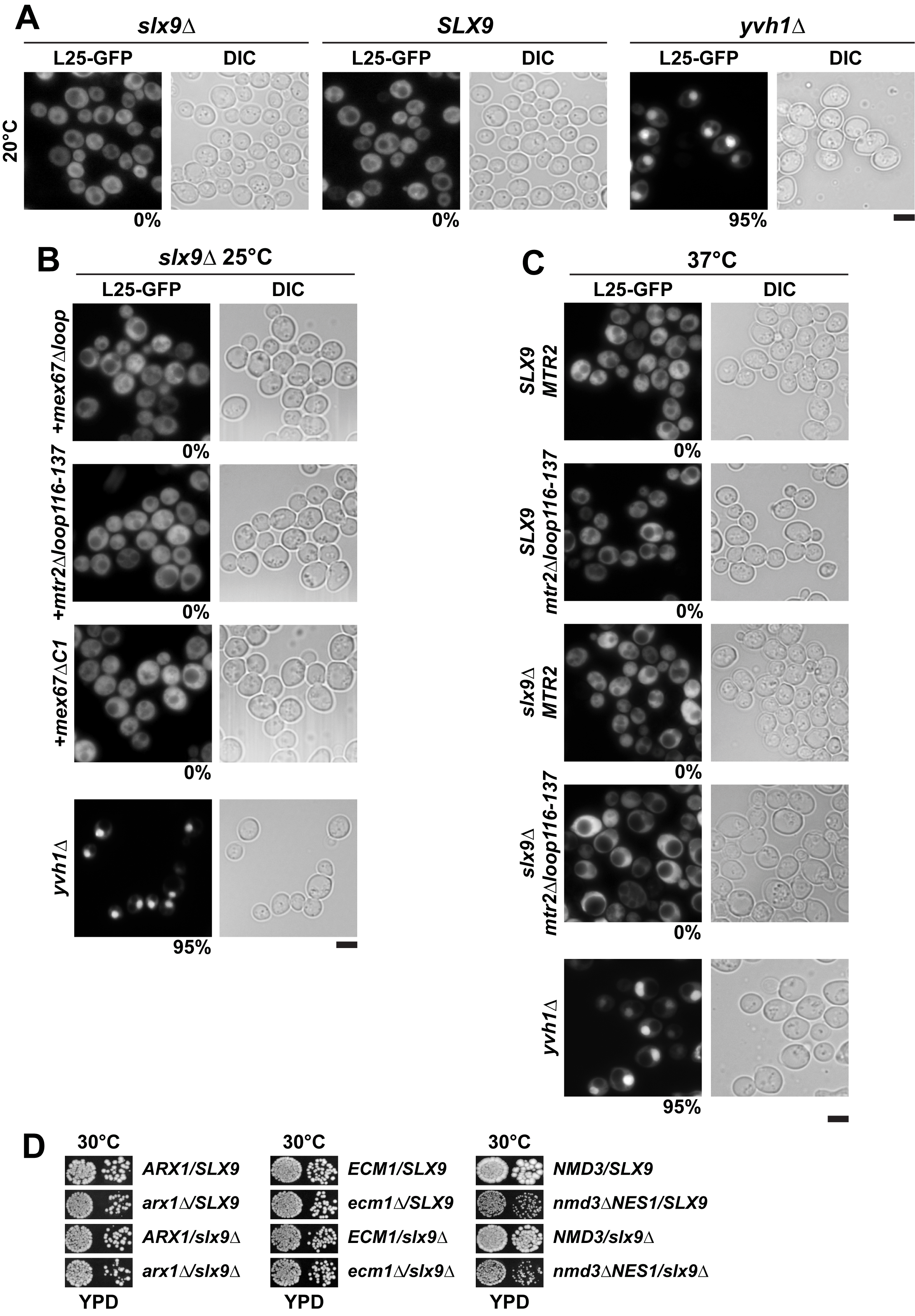

Supplement: Figure S3 — (A) The slx9Δ mutant is not impaired in pre60S subunit nuclear export. SLX9 and slx9Δ strains containing the 60S reporter, L25-GFP, were grown at 20°C and inspected by fluorescence microscopy. Percentage of cells that showed nuclear accumulation of the L25-GFP is indicated below each picture panel. The yvh1Δ mutant that accumulates the L25-GFP in the nucleoplasm in the temperature range between 20–37°C served as positive control. Bar = 5 µm. (B) Expression of mex67Δloop, mex67ΔC1 and mtr2Δloop116-137 alleles in the slx9Δ mutant does not induce a pre60S subunit export defect. The slx9Δ mutant containing the L25-GFP reporter was transformed with the indicated plasmids and grown at 25°C. The localization of L25-GFP was inspected by fluorescence microscopy. Percentage of cells showing nuclear accumulation of the L25-GFP is indicated below each picture panel. The yvh1Δ mutant that accumulates the L25-GFP in the nucleoplasm at 25°C served as positive control. Bar = 5 µm. (C) The synthetically enhanced slx9Δmtr2Δloop116-137 strain is not impaired in pre60S subunit export. Localization of L25-GFP in the indicated strains was inspected by fluorescence microscopy at 37°C. Percentage of cells showing nuclear accumulation of the L25-GFP is indicated below each picture panel. The yvh1Δ mutant that accumulates the L25-GFP in the nucleoplasm at 37°C served as positive control. Bar = 5 µm. (D) Slx9 does not genetically interact with factors involved in pre60S subunit export. Growth of the slx9Δ mutant combined with the arx1Δ, ecm1Δ and the nmd3ΔNES1 mutants. The indicated strains were spotted in 10-fold serial dilutions on YPD plates and grown at 30°C for 2–3 days. (TIF) [file pgen.1002915.s003.tif]

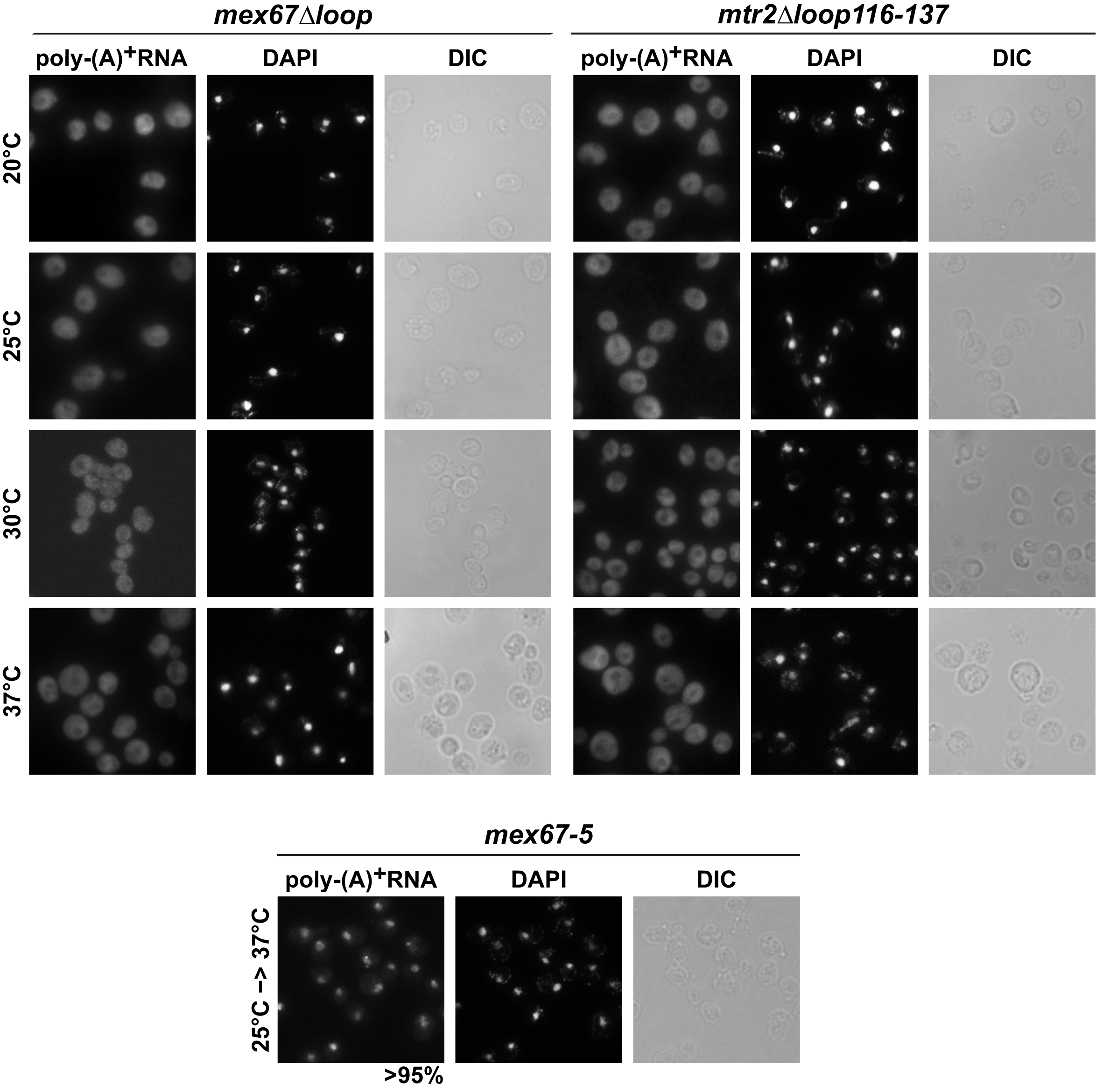

Supplement: Figure S4 — The mex67Δloop and mtr2Δloop116-137 alleles do not accumulate poly-(A)+RNA in the nucleus. The mex67Δloop and mtr2Δloop116-137 strains were grown at the indicated temperatures. Localization of poly-(A)+RNA was performed by FISH using Cy3-oligo-(dT)30. Nuclear and mitochondrial DNA was stained with DAPI. The mex67-5 strain that accumulated poly-(A)+RNA at 37°C served as positive control. The mex67-5 strain was grown at 25°C, then shifted to 37°C for 1 h prior to analyses. Percentage of cells showing nuclear accumulation of poly-(A)+RNA is indicated below each picture panel. Bar = 5 µm. (TIF) [file pgen.1002915.s004.tif]

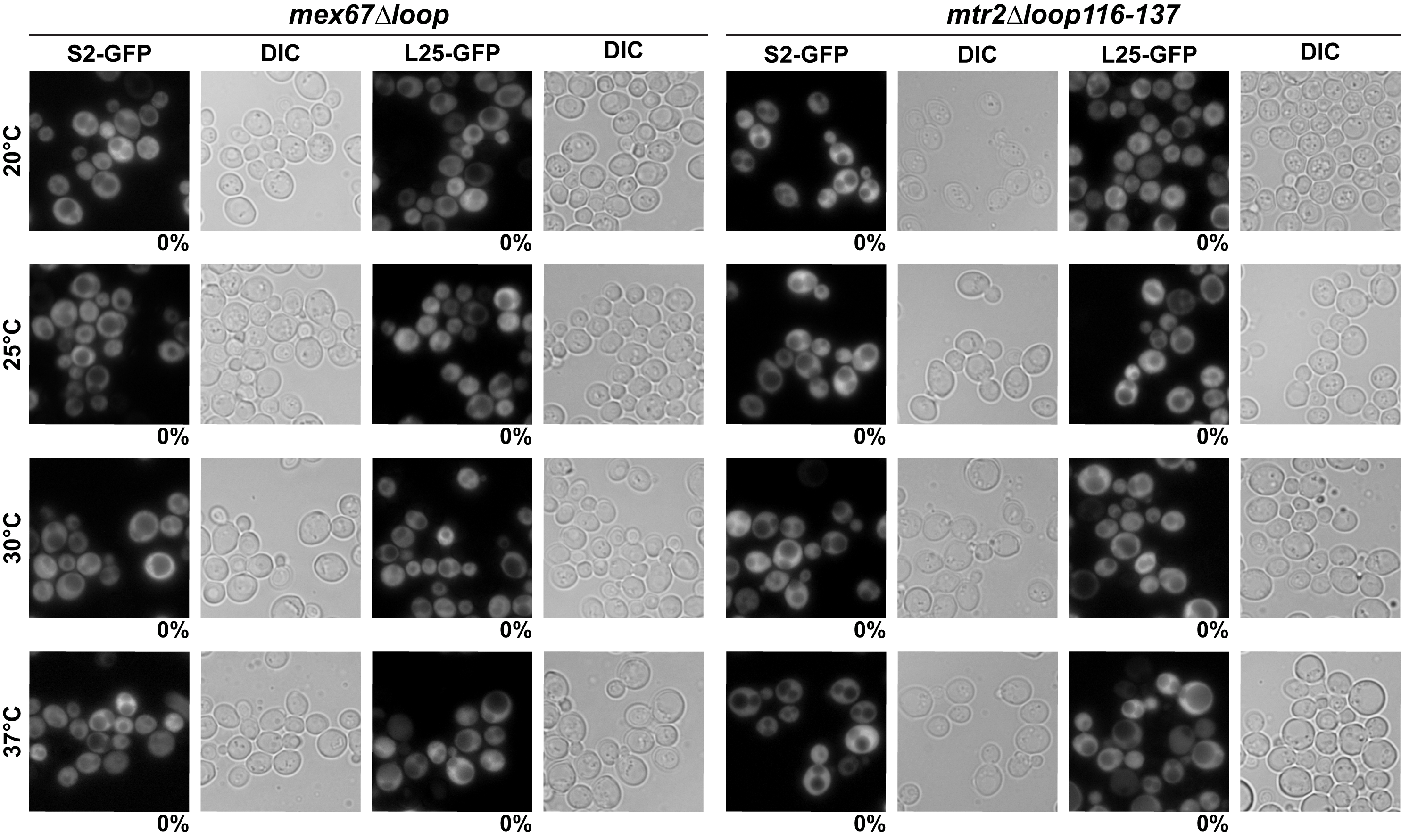

Supplement: Figure S5 — The mex67Δloop and mtr2Δloop116-137 alleles are not impaired in pre40S and pre60s subunit nuclear export. The mex67Δloop and mtr2Δloop116-137 strains containing S2-GFP or L25-GFP were grown at the indicated temperatures and inspected by fluorescence microscopy. Percentage of cells showing nuclear accumulation of the S2-GFP and L25-GFP is indicated below each picture panel. Bar = 5 µm. (TIF) [file pgen.1002915.s005.tif]

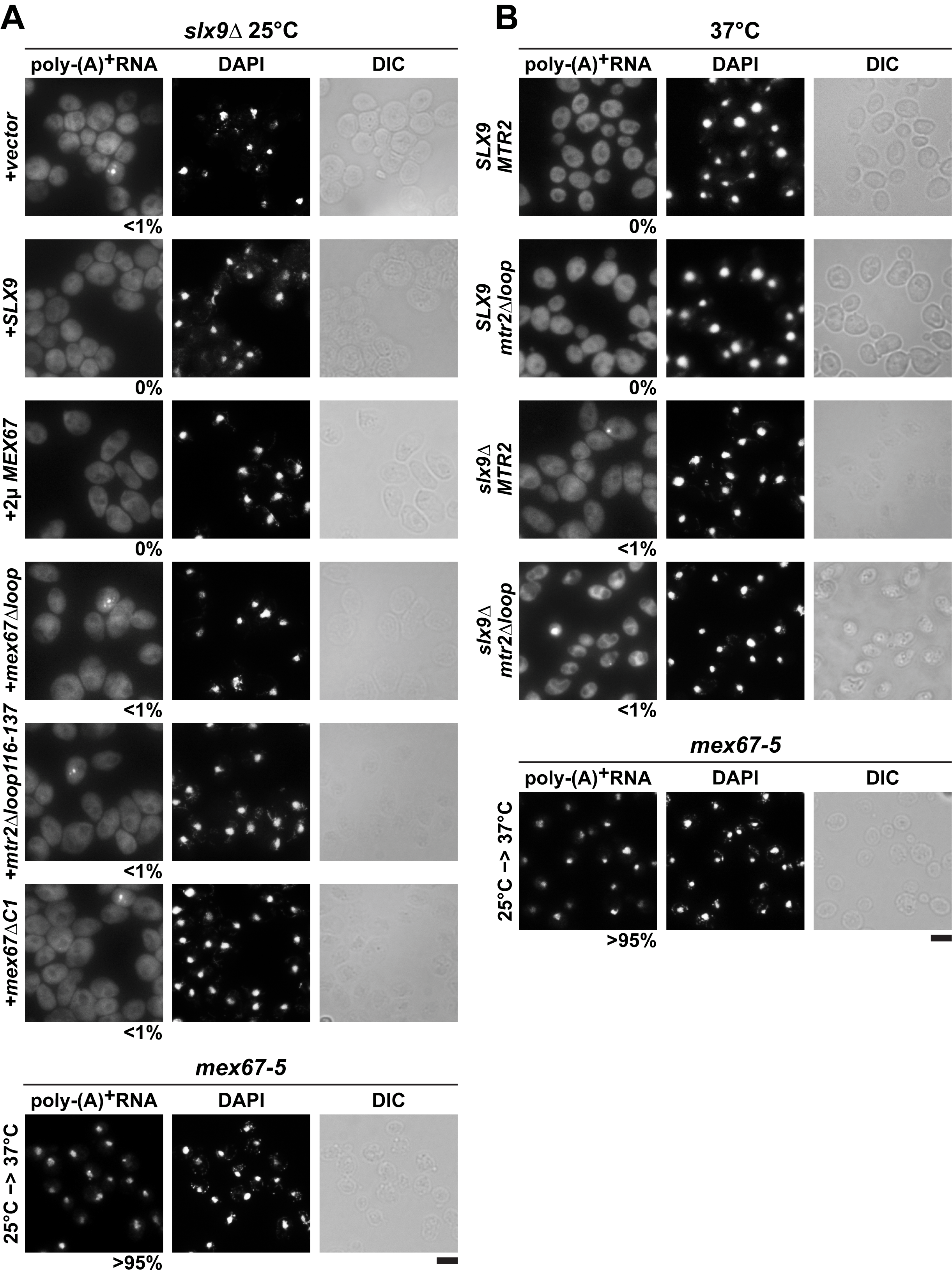

Supplement: Figure S6 — (A) Expression of mex67Δloop, mex67ΔC1 and mtr2Δloop116-137 alleles does not exacerbate nuclear accumulation of poly-(A)+RNA in the slx9Δ mutant. The slx9Δ mutant containing mex67Δloop, mex67ΔC1 and mtr2Δloop116-137 alleles were grown at 25°C to mid-log phase. Localization of poly-(A)+RNA was performed by FISH using Cy3-labelled oligo-(dT)30. Nuclear and mitochondrial DNA was stained with DAPI. Percentage of cells showing nuclear accumulation of poly-(A)+RNA is indicated below each picture panel. The mex67-5 strain that accumulated poly-(A)+RNA at 37°C served as positive control. The mex67-5 strain was grown at 25°C, then shifted to 37°C for 1 h prior to analyses. Percentage of cells that showed nuclear accumulation of poly-(A)+RNA is indicated below each picture panel. Bar = 5 µm. (B) Nuclear accumulation of poly-(A)+RNA is not aggravated in the synthetically enhanced slx9Δmtr2Δloop116-137 strain. The indicated strains were grown to mid-log phase at 37°C. Localization of poly-(A)+RNA was performed by FISH using Cy3-labelled oligo-(dT)30. Nuclear and mitochondrial DNA was stained with DAPI. Percentage of cells showing nuclear accumulation of poly-(A)+RNA is indicated below each picture panel. The mex67-5 strain that accumulated poly-(A)+RNA at 37°C served as positive control. The mex67-5 strain was grown at 25°C, then shifted to 37°C for 1 h prior to analyses. Percentage of cells that showed nuclear accumulation of poly-(A)+RNA is indicated below each picture panel. Bar = 5 µm. (TIF) [file pgen.1002915.s006.tif]

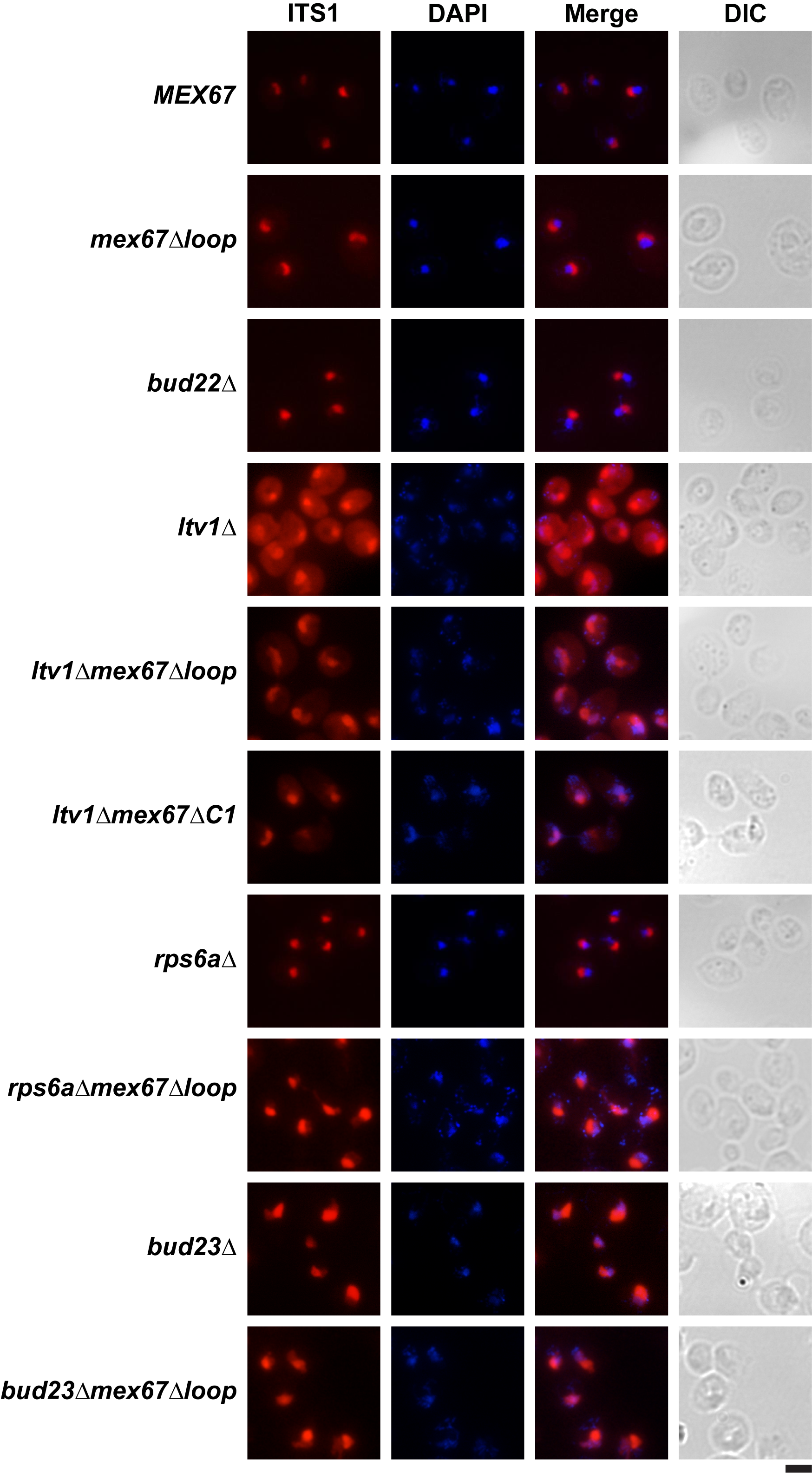

Supplement: Figure S7 — Synthetically enhanced double mutant strains accumulate ITS1 in the nucleoplasm. The indicated strains analysed in Figure 8 were grown to mid-log phase at 30°C and shifted to 20°C for 3 h. Localization of 20S rRNA was analysed by FISH using a Cy3-labeled oligonucleotide complementary to the 5′ portion of ITS1 (red). Nuclear and mitochondrial DNA was stained with DAPI (blue). Bar = 5 µm. (TIF) [file pgen.1002915.s007.tif]
