# Supplementary material for: Role of Mex67-Mtr2 in the Nuclear Export of 40S Pre-Ribosomes
Source: PLoS Genet. 2012 Aug 30;8(8):e1002915. doi: 10.1371/journal.pgen.1002915 (PMC3431309; doi:10.1371/journal.pgen.1002915)
Supplement: Table S1 — List of plasmids used in this study. (PDF) [file pgen.1002915.s008.pdf]

Table S1. **Plasmids used in this study**

**Plasmid**	**Relevant markers**	**Source**
pRS316-S2-eGFP	RPS2-eGFP CEN URA3	[1]
pRS314-SLX9	SLX9 CEN TRP1	this study
pRS315-SLX9	SLX9 CEN LEU2	this study
pRS316-SLX9	SLX9 CEN URA3	this study
pRS426-MEX67	MEX67 2µ URA3	[2]
pUN100-mex67-5	mex67(H400Y) CEN LEU2	[3]
pRS426-MTR2	MTR2 2µ URA3	[2]
pUN100-ARX1	ARX1 CEN LEU2	this study
pRS314-ECM1	ECM1 CEN TRP1	[4]
pRS316-NMD3	NMD3 CEN URA3	[5]
pRS426-RRP12	RRP12 2µ URA3	this study
pRS315-mex67ΔC1	mex67(Δ525-599aa) CEN LEU2	[6]
pRS314-mex67Δloop	mex67(Δ409-435aa) CEN TRP1	[7]
pRS314-mtr2Δloop116-137	mtr2(Δ116-137aa) CEN TRP1	[7]
pRS316-L25-eGFP	RPL25-GFP CEN URA3	[8]
pRS314-nmd3ΔNES1	nmd3ΔNES1 CEN TRP1	[7]
pUN100-SUB2	SUB2 CEN LEU2	[9]
pRS315-sub2-85	sub2-85 CEN LEU2	[9]
pUN100-YRA1	YRA1 CEN LEU2	[10]
pRS315-yra1ΔRRM	yra1ΔRRM CEN LEU2	[10]
pRS315-YVH1	YVH1 CEN LEU2	[11]
pRS313-XPO1	XPO1 CEN HIS3	[12]
pRS313-xpo1-1	xpo1-1 CEN HIS3	[12]

1. Milkereit P, Strauss D, Bassler J, Gadal O, Kühn H, et al. (2003) A Noc complex specifically involved in the formation and nuclear export of ribosomal 40 S subunits. J Biol Chem 278: 4072-4081.

2. Hung N-J, Lo K-Y, Patel SS, Helmke K, Johnson AW (2008) Arx1 is a nuclear export receptor for the 60S ribosomal subunit in yeast. Mol Biol Cell 19: 735-744.

3. Segref A, Sharma K, Doye V, Hellwig A, Huber J, et al. (1997) Mex67p, a novel factor for nuclear mRNA export, binds to both poly(A)+ RNA and nuclear pores. EMBO J 16: 3256-3271.

4. Baßler J, Grandi P, Gadal O, Leßmann T, Petfalski E, et al. (2001) Identification of a 60S Preribosomal Particle that Is Closely Linked to Nuclear Export. Mol Cell 8: 517-529.

5. Gadal O, Strauß D, Kessl J, Trumpower B, Tollervey D, et al. (2001) Nuclear export of 60S ribosomal subunits depends on Xpo1p and requires a nuclear export sequence-containing

factor, Nmd3p, that associates with the large subunit protein Rpl10p. Mol Cell Biol 21: 3405-3415.

6. Sträßer K, Baßler J, Hurt E (2000) Binding of the Mex67p/Mtr2p heterodimer to FXFG, GLFG, and FG repeat nucleoporins is essential for nuclear mRNA export. J Cell Biol 150: 695-706.

7. Yao W, Roser D, Köhler A, Bradatsch B, Baßler J, et al. (2007) Nuclear export of ribosomal 60S subunits by the general mRNA export receptor Mex67-Mtr2. Mol Cell 26: 51-62.

8. Hurt E, Hannus S, Schmelzl B, Lau D, Tollervey D, et al. (1999) A novel in vivo assay reveals inhibition of ribosomal nuclear export in Ran-cycle and nucleoporin mutants. J Cell Biol 144: 389-401.

9. Sträßer K, Hurt E (2001) Splicing factor Sub2p is required for nuclear mRNA export through its interaction with Yra1p. Nature 413: 648-652.

10. Sträßer K, Masuda S, Mason P, Pfannstiel J, Oppizzi M, et al. (2002) TREX is a conserved complex coupling transcription with messenger RNA export. Nature 417: 304-308.

11. Kemmler S, Occhipinti L, Veisu M, Panse VG (2009) Yvh1 is required for a late maturation step in the 60S biogenesis pathway. J Cell Biol 186: 863-880.

12. Bradatsch B, Katahira J, Kowalinski E, Bange G, Yao W, et al. (2007) Arx1 functions as an unorthodox nuclear export receptor for the 60S preribosomal subunit. Mol Cell 27: 767-779.
