# Supplementary material for: Role of Mex67-Mtr2 in the Nuclear Export of 40S Pre-Ribosomes
Source: PLoS Genet. 2012 Aug 30;8(8):e1002915. doi: 10.1371/journal.pgen.1002915 (PMC3431309; doi:10.1371/journal.pgen.1002915)
Supplement: Table S2 — List of yeast strains used in this study. (PDF) [file pgen.1002915.s009.pdf]

Table S2. **Yeast strains used in this study**

**Strain name**	**Genotype**	**Origin**
BY4741	*MATa ura3 his3 leu2 met15 TRP1*	Euroscarf
Arx1-TAP	*MATα his3 leu2 ura3 Arx1-TAP::TRP1*	[1]
Enp1-TAP	*MATa ura3 his3 leu2 TRP1 Enp1-TAP::HIS3MX*	Open biosystems
Enp1-TAP *mex67* shuffle	*MATα ura3 his3 leu2 ade2 TRP1 mex67::HIS3MX ENP1-TAP::HIS3MX <pRS316-MEX67>*	this study
Enp1-TAP *mtr2* shuffle	*MATα ura3 his3 leu2 ade2 TRP1 mtr2::HIS3MX ENP1-TAP::HIS3MX <pRS316-MTR2>*	this study
Enp1-TAP *slx9Δ*	*MATa ura3 his3 leu2 TRP1 Enp1-TAP::HIS3MX slx9:: NATNT2*	this study
Hrr25-TAP	*MATa ura3 his3 leu2 TRP1 Hrr25-TAP::HIS3MX*	Open biosystems
Kre35-TAP	*MATα ura3 his3 leu2 trp1 met15 Kre35-TAP::TRP1*	[1]
mex67 shuffle *slx9Δ*	*MATa ura3 his3 leu2 trp1 ade2 mex67::HIS3MX slx9::KANMX <pRS316-MEX67>*	this study
mex67 shuffle *yrb2Δ*	*MATa ura3 his3 leu2 trp1 ade2 mex67::HIS3MX <pRS316-MEX67> yrb2::KAN*	this study
mex67 shuffle *rps15-1*	*MATa ura3 his3 leu2 trp1 ade2 mex67::HIS3MX <pRS316-MEX67> rps15::KAN <pRS316-MEX67> <Rps15-LEU>*	this study
mex67 shuffle *bud23Δ*	*MATa ura3 his3 leu2 ade2 mex67::HIS3MX bud23::KANMX <pRS316-MEX67>*	this study
mex67 shuffle *rps6aΔ*	*MATa ura3 his3 leu2 ade2 mex67::HIS3MX rps6a::KANMX <pRS316-MEX67>*	this study
mex67 shuffle *ltv1Δ*	*MATa ura3 his3 leu2 trp1 ade2 mex67::HIS3MX ltv1::KAN <pRS316-MEX67>*	this study
mtr2 shuffle *slx9Δ*	*MATa ura3 his3 leu2 trp1 ade2 mtr2::HIS3MX slx9::KANMX <pRS316-MTR2>*	this study
mtr2 shuffle *yrb2Δ*	*MATa ura3 his3 leu2 trp1 ade2 mtr2::HIS3MX yrb2::KAN<pRS316-MTR2>*	this study
mtr2 shuffle *rps15-1*	*MATa ura3 his3 leu2 trp1 ade2 mtr2::HIS3MX rps15::KAN <pRS316-MTR2> <Rps15-LEU>*	this study
mtr2 shuffle *bud23Δ*	*MATa ura3 his3 leu2 ade2 mtr2::HIS3MX bud23::KANMX <pRS316-MTR2>*	this study

mtr2 shuffle *rps6a*Δ	*MATalpha ura3 his3 leu2 ade2 mtr2::HIS3MX rps6a::KANMX <pRS316-MTR2>*	this study
mtr2 shuffle *ltv1*Δ	*MATa ura3 his3 leu2 trp1 ade2 mtr2::HIS3MX ltv1::KAN <pRS316-MTR2>*	this study
nmd3 shuffle *slx9*Δ	*MATa his3 leu2 ura3 trp1 slx9::HIS3MX nmd3::KANMX4 <pRS316-NMD3>*	this study
Noc4-TAP	*MATa ura3 his3 leu2 TRP1 Noc4-TAP::HIS3MX*	Open biosystems
Rio2-TAP	*MATa ura3 his3 leu2 TRP1 Rio2-TAP::HIS3MX*	Open biosystems
Rio2-TAP *mex67* shuffle	*MATa ura3 his3 leu2 trp1 ade2 mex67::HIS3MX Rio2-TAP::KANMX <pRS316-MEX67>*	this study
Rio2-TAP *mtr2* shuffle	*MATa ura3 his3 leu2 trp1 ade2 mtr2::HIS3MX Rio2-TAP::KANMX <pRS316-MTR2>*	this study
Rio2-TAP *yrb2*Δ	*MATa; his3D1; leu2D0; met15D0; yrb2::KANMX RIO2-TAP::URA*	this study
Rix1-TAP	*MATα ura3 his3 leu2 trp1 met15 Rix1-TAP::TRP1*	[1]
GAL-rps15-1	*MATα his3 leu2 ura3 rps15::KANMX <pFL36-pGal-rps15-1>*	[2]
Slx9-GFP Gar1-mCherry	*MATa/MATα ura3 his3 leu2 met15 TRP1 slx9GFP::HIS3MX Gar1mCherry::KANMX*	this study
*slx9*Δ	*MATα ura3 his3 leu2 TRP1 slx9::KANMX*	this study
*slx9*Δ	*MATa ura3 his3 leu2 TRP1 slx9::HIS3MX*	this study
*slx9*Δ *arx1*Δ	*MATa ura3 his3 leu2 TRP1 slx9:: NATNT2 arx1::KANMX*	this study
*slx9*Δ *ecm1*Δ	*MATa ura3 his3 leu2 TRP1 slx9::NATNT2 ecm1::KANMX*	this study
*slx9*Δ *sub2* shuffle	*MATα ura3 his3 leu2 TRP1 slx9::KANMX sub2::KAN<pRS316-SUB2>*	this study
*slx9*Δ *yra1* shuffle	*MATa ura3 his3 leu2 TRP1 slx9::KANMX yra1::HIS3<pRS316-YRA1>*	this study
Ssf1-TAP	*MATα his3 leu2 ura3 Ssf1-TAP ::TRP1*	[1]
xpo1-1	*MATα ura3 his3 leu2 ade2 trp1 can1 xpo1::LEU2 <pKW457-xpo1-1-HIS3>*	[3]
*yvh1*Δ	*MATa ura3 his3 leu2 TRP1 yvh1::KANMX*	[4]

1. Yao W, Roser D, Köhler A, Bradatsch B, Baßler J, et al. (2007) Nuclear export of ribosomal 60S subunits by the general mRNA export receptor Mex67-Mtr2. Mol Cell 26: 51-62.

2. Léger-Silvestre I, Milkereit P, Ferreira-Cerca S, Saveanu C, Rousselle J-C, et al. (2004) The ribosomal protein Rps15p is required for nuclear exit of the 40S subunit precursors in yeast. EMBO J 23: 2336-2347.

3. Stade K, Ford CS, Guthrie C, Weis K (1997) Exportin 1 (Crm1p) is an essential nuclear export factor. Cell 90: 1041-1050.

4. Kemmler S, Occhipinti L, Veisu M, Panse VG (2009) Yvh1 is required for a late maturation step in the 60S biogenesis pathway. J Cell Biol 186: 863-880.
